# Loss of Multiple ABCB Auxin Transporters Recapitulates the Major *twisted dwarf 1* Phenotypes in *Arabidopsis thaliana*

**DOI:** 10.3389/fpls.2022.840260

**Published:** 2022-04-21

**Authors:** Mark K. Jenness, Reuben Tayengwa, Gabrielle A. Bate, Wiebke Tapken, Yuqin Zhang, Changxu Pang, Angus S. Murphy

**Affiliations:** ^1^Department of Plant Science and Landscape Architecture, University of Maryland, College Park, MD, United States; ^2^School of Plant Sciences and Food Security, Tel Aviv University, Tel Aviv, Israel

**Keywords:** ABCB transporter, auxin, organ twisting, FKBP42/TWD1, *Arabidopsis thaliana*

## Abstract

FK506-BINDING PROTEIN 42/TWISTED DWARF 1 (FKBP42/TWD1) directly regulates cellular trafficking and activation of multiple ATP-BINDING CASSETTE (ABC) transporters from the ABCB and ABCC subfamilies. *abcb1 abcb19* double mutants exhibit remarkable phenotypic overlap with *twd1* including severe dwarfism, stamen elongation defects, and compact circinate leaves; however, *twd1* mutants exhibit greater loss of polar auxin transport and additional helical twisting of roots, inflorescences, and siliques. As *abcc1 abcc2* mutants do not exhibit any visible phenotypes and TWD1 does not interact with PIN or AUX1/LAX auxin transporters, loss of function of other ABCB auxin transporters is hypothesized to underly the remaining morphological phenotypes. Here, gene expression, mutant analyses, pharmacological inhibitor studies, auxin transport assays, and direct auxin quantitations were used to determine the relative contributions of loss of other reported ABCB auxin transporters (4, 6, 11, 14, 20, and 21) to *twd1* phenotypes. From these analyses, the additional reduction in plant height and the twisted inflorescence, root, and silique phenotypes observed in *twd1* compared to *abcb1 abcb19* result from loss of *ABCB6* and *ABCB20* function. Additionally, *abcb6 abcb20* root twisting exhibited the same sensitivity to the auxin transport inhibitor 1-napthalthalamic acid as *twd1* suggesting they are the primary contributors to these auxin-dependent organ twisting phenotypes. The lack of obvious phenotypes in higher order *abcb4* and *abcb21* mutants suggests that the functional loss of these transporters does not contribute to *twd1* root or shoot twisting. Analyses of ABCB11 and ABCB14 function revealed capacity for auxin transport; however, their activities are readily outcompeted by other substrates, suggesting alternate functions *in planta*, consistent with a spectrum of relative substrate affinities among ABCB transporters. Overall, the results presented here suggest that the ABCB1/19 and ABCB6/20 pairs represent the primary long-distance ABCB auxin transporters in Arabidopsis and account for all reported *twd1* morphological phenotypes. Other ABCB transporters appear to participate in highly localized auxin streams or mobilize alternate transport substrates.

## Introduction

A unique group of FK506-BINDING PROTEINs (FKBPs) function in the folding of ATP-BINDING CASSETTE (ABC) transporter proteins (Geisler et al., 2003, 2004; Banasavadi-Siddegowda et al., 2011). These “long” FKBPs contain between one and three FK506-binding domains (FKBD), a tetratricopeptide repeat (TPR) domain, a calmodulin binding domain (CaM-BD), and an in-plane membrane anchor (IPM) and are found ubiquitously in eukaryotes (Geisler and Bailly, 2007). A prominent example is mammalian FKBP8/38 that functions in the post-translational folding of the ATP-gated chloride channel ABCC7/Cystic Fibrosis Transmembrane Conductance Regulator (CFTR) on the endoplasmic reticulum (ER) surface to promote trafficking to the plasma membrane (PM; Banasavadi-Siddegowda et al., 2011; Hutt et al., 2012).

In Arabidopsis, FK506-BINDING PROTEIN 42 (FKBP42) functions in the processing of ATP-BINDING CASSETTE subfamily B (ABCB) and C (ABCC) transporters at the ER and is required for localization and subsequent functionality of these proteins on the plasma and vacuolar membranes, respectively (Geisler et al., 2003, 2004; Bouchard et al., 2006; Bailly et al., 2008; Wu et al., 2010; Wang et al., 2013). Loss of FKBP42 underlies the pronounced phenotypes of the Arabidopsis *twisted dwarf 1* (*twd1*) mutant including dwarfism, stamen defects and reduced fertility, and compact circinate leaves, as well as helical twisting of roots, inflorescences, and siliques (Pérez-Pérez et al., 2002; Geisler et al., 2003). In roots, *twd1* helical cell file twisting is non-handed, occurring in both left- and right-handed orientations (Weizbauer et al., 2011; Wang et al., 2013). In *Medicago trunculata*, loss of the TWD1 ortholog SSP1 also results in defective auxin-dependent seedpod spine formation (Zhao et al., 2021).

Initial molecular interaction studies using yeast two-hybrid identified ABCB1/19 and ABCC1/2 as strong TWD1 interactors (Geisler et al., 2003, 2004). Subsequent studies using fluorescent protein fusions and bioluminescence resonance energy transfer (BRET) validated these interactions for ABCB1/19 and identified ABCB4 as an additional TWD1 interactor (Wu et al., 2010; Kubeš et al., 2012; Wang et al., 2013; Hao et al., 2020). These reports showed that TWD1 functions at the ER and is required for trafficking and activity of the most abundant ABCB isoforms (1, 4, and 19) at the PM. TWD1 interactions with the C-termini of ABCB and ABCC protein subfamily members are mechanistically distinct, as ABCBs associate with the FKBP domain of TWD1 while ABCCs interact with a distinct tetratricopeptide repeat domain (Geisler et al., 2003, 2004). Additional direct and genetic associations of TWD1 with other proteins including PINOID (Henrichs et al., 2012), ACTIN7 (Zhu et al., 2016), and BRI1 (Chaiwanon et al., 2016; Zhao et al., 2016) have also been identified.

Several *twd1* phenotypes are also observed in mutants lacking functional ABCB auxin transporters. *abcb1 abcb19* double mutants (hereafter *abcb1/19*) also exhibit severe dwarf stature, circinate leaves, and reduced auxin-dependent stamen elongation and dehiscence defects associated with reduced seed set (Noh et al., 2001; Blakeslee et al., 2007; Titapiwatanakun et al., 2009; Cecchetti et al., 2015). However, helical twisting of roots, stems, and siliques in *twd1* are not observed in *abcb1/19*. Despite the strong interaction of TWD1 with ABCC1 and ABCC2, *abcc1 abcc2* double mutants do not show any obvious morphological defects (Geisler et al., 2004; Song et al., 2010). Therefore, a compelling rationale for the phenotypic differences between *twd1* and *abcb1/19* is the activation of other ABCB auxin transporters by TWD1 (Geisler et al., 2017). This is corroborated by the additional ~10–15% loss in rootward auxin transport in *twd1* compared to *abcb1/19* (Geisler et al., 2003). However, *abcb* mutational analyses are complicated by multiple gene duplication events observed in the plant *ABCB* gene family (Verrier et al., 2008; Knöller et al., 2010; Kang et al., 2011; Banasiak and Jasiński, 2021). As a result, any mutational analysis of *ABCB* transporters must consider overlapping function and sometimes compensatory expression of highly similar gene products, most notably Arabidopsis *ABCB1/19*, *4/21*, *6/20*, 13/14, and *11/12* (Noh et al., 2001; Geisler et al., 2003; Lin and Wang, 2005; Kaneda et al., 2011; Kamimoto et al., 2012; Zhang et al., 2018; Jenness et al., 2019). High degrees of sequence similarity and linkage disequilibria resulting from gene duplication with *ABCB* gene clusters further complicate mutational and induced loss of function analyses. Further, light regimes appear to regulate TWD1 activation of ABCBs (Christie et al., 2011).

*TWD1* is ubiquitous, but abundance is relatively low, with typical transcript levels <10% of *ABCB1* and *ABCB19* combined (Geisler et al., 2003; Klepikova et al., 2016). The observed stoichiometry is consistent with proposed and experimentally demonstrated activities of TWD1 (Geisler et al., 2016), the limited increases in auxin transport activity observed with TWD1 and ABCB combined overexpression (Bouchard et al., 2006), and evidence that only overexpression of TWD1 without its hydrophobic membrane association domain leads to increased auxin transport and enhanced growth due to increased ABCB activity (Bailly et al., 2013). Cumulatively, these reports also suggest that any loss of additional ABCB transporter function associated with *twd1* phenotypes occurs in discrete tissues where the specific isoforms are more abundant.

Plant ABCB transporters appear to be selective for aromatic and aliphatic organic acids, with enhanced specificity evident when ABCBs, PINs, and TWD1 co-occur (Geisler et al., 2005; Bouchard et al., 2006; Titapiwatanakun et al., 2009; Yang and Murphy, 2009; Bailly et al., 2012, 2013; Deslauriers and Spalding, 2021). Recently, a C-terminal proline residue (P1008 in ABCB1) present in a subgroup of ABCB transporters that have been associated with auxin transport (ABCB1, 19, 6, 20, 4, 21, 15–18, 22) was identified (Hao et al., 2020). The authors imply that this residue is an identifier for ABCBs that may be activated by the peptidyl-prolyl *cis*-*trans* isomerase (PPIase) activity of TWD1 and consequent specificity for auxin efflux. However, the importance of this proline residue is equivocal since attempts to demonstrate TWD1 PPIase activity have been unsuccessful (Geisler et al., 2003), and the conserved proline residue was not essential for ABCB1-TWD1 interaction (Hao et al., 2020).

Systematic biochemical characterization of TWD1 interactions with the full range of 21 Arabidopsis ABCB transporters has been limited by intrinsic qualities of the ABCB proteins. Some ABCB proteins have proven to be non-functional or highly mis-localized when expressed in commonly used mammalian cell systems, tobacco BY-2 cells, or *Saccharomyces cerevisiae* (Noh et al., 2001; Geisler et al., 2005; Kubeš et al., 2012). As a result, yeast two-hybrid assays can only be performed with soluble protein fragments (Geisler et al., 2003). Other plant and animal cell systems previously used to study the auxin transport activity of some ABCBs (Petrášek et al., 2006; Blakeslee et al., 2007) have proven to be impractical for systematic protein interaction studies. A BRET-based system has been used to study TWD1 interactions with some ABCBs, but has not been useful for evaluation of Arabidopsis ABCB19 (Bailly et al., 2008; Wang et al., 2013; Hao et al., 2020). Partially functional ABCB-fluorescent protein fusions have been used to demonstrate cellular trafficking defects of ABCB1, 4, and 19 (Wu et al., 2010; Wang et al., 2013; Yang et al., 2013). However, application of this methodology to low abundance ABCB members has limited utility, as overexpression invariably results in distributions in the endomembrane compartments observed when trafficking of ABCB19 is perturbed (Yang et al., 2013).

A similar limitation has been observed in comparative proteomic studies. Arabidopsis ABCBs are not amenable to many standard protein biochemistry techniques (Noh et al., 2001; Terasaka et al., 2005; Blakeslee et al., 2007; Titapiwatanakun et al., 2009; Yang and Murphy, 2009), and only abundant isoforms are reported in proteomic identifications of PM fractions or interaction studies if present at all (Alexandersson et al., 2004; Borner et al., 2005; Titapiwatanakun et al., 2009; Henrichs et al., 2012; Demir et al., 2013; Zhu et al., 2016). As a result, differential distributions of less abundant ABCBs between *twd1* and Col-0 PMs are not convincing. However, affinity chromatography using the ABCB/TWD1 binding auxin efflux inhibitor 1-naphthylphthalamic acid (NPA) identified ABCB1, 4, and 19 as well as at least two other unresolved ABCBs when analyzed by mass spectroscopy (Bernasconi et al., 1996; Butler et al., 1998; Murphy et al., 2002).

Here we engaged in a phenotypic analysis of known and putative ABCB auxin transporters under uniform conditions and a single ecotypic background to ascertain that all the major phenotypes associated with *twd1* are observed with combined loss of four ABCB transporters. Analysis of *ABCB* transporter expression identified *ABCB1/19*, and *ABCB6*/*20* as the primary candidates underlying *twd1* phenotypes in aerial tissues and *ABCB1/19, 4/21, 6/20*, and *11* in the root. Mutant analysis showed that combined loss of ABCB1/19, and ABCB6/20 function is sufficient to confer all the major phenotypes associated with *twd1*. Further investigation reveals ABCB11 and ABCB14 can transport auxin but likely have alternate substrates *in planta*.

## Materials and Methods

### Plant Material and Growth Conditions

Mutants used in this study were in the Col-0 background and previously described: *abcb1-100* (Lin and Wang, 2005), *abcb19-101* (Lin and Wang, 2005), *abcb1-100 abcb19-101* (Jenness et al., 2019), *abcb4-1* (Terasaka et al., 2005), *abcb21-2* (Jenness et al., 2019), *abcb4-1 abcb21-2* (Jenness et al., 2019), *amiRNA1334* (Zhang et al., 2018), *abcb6-1* (Zhang et al., 2018), *abcb20-1* (Zhang et al., 2018), *abcb20-2* (Zhang et al., 2018), *abcb6-1 abcb20-2* (Zhang et al., 2018), *twd1-3* (Geisler et al., 2003), *abcb11-1* (SALK_057628; this study), *abcb11-2* (SALK_037942; this study), *abcb14-1* (Kaneda et al., 2011), *pin1-7* (Furutani et al., 2004). All triple mutants were generated by crossing the alleles designated in the text and figure legends into *abcb1-100 abcb19-101*. Specific alleles in each figure and table are listed in Supplementary Table 3. For seedling growth, surface-sterilized seeds were plated on ¼ MS medium containing 1 g L^−1^ MES, 0.5% sucrose, and 0.8% agar (pH 5.6). Seeds were stratified 2 days in the dark at 4°C, then grown vertically under continuous 100 μmol m^−2^ s^−1^ light at 22°C for the times indicated. For mature plants, seeds were sown on soil and stratified 2–4 days in the dark at 4°C. Plants were grown in growth chambers with fluorescent light supplemented with incandescent bulbs set to 100 μmol m^−2^ s^−1^, 16 h photoperiod, and 21°C for the times specified.

### Seedling and Mature Plant Imaging and Measurements

Images of mature plants were taken with a Cannon Stylus 1010 digital camera set on a tripod for stability. Close-up leaf, inflorescence, and silique images were collected on a Zeiss Stemi-2000 stereo microscope (Carl Zeiss, Germany) using Lumenera Infinity2 software (Lumenera Corp.). Seedling and root cell file images were collected on a Leica DMI6000B using LAS imaging software (Leica Co., Germany) with the exception of *amiRNA1334* and *abcb6-1 abcb20-1* which were collected on a Zeiss LSM 780 using a 10× objective. Root length measurements were made from high-resolution scanned images. All seedling measurements were made using ImageJ (Schneider et al., 2012). Measurements for mature aerial tissues were done manually. Brightness and contrast were adjusted using Adobe Photoshop to enhance visualization of the cell profiles equally within each image set as noted in the respective figure legends. All assays were conducted at least three times with similar results.

### Quantitative Real-Time PCR

Total RNA was extracted using ZR Plant RNA Mini Prep kit (Zymo Research) followed by treatment with DNaseI (New England Biolabs). Total RNA (1.5 μg) was used for first-strand synthesis using SuperScript III reverse transcriptase (Thermo Fisher Scientific). Real-time PCR was performed on a CFX Connect (Bio-Rad Laboratories) using EvaGreen qPCR master mix (Biotium). Primers used are listed in Supplementary Table 1. Transcript levels normalized against PP2A (AT1G69960) or ACT2 (AT3G18780) produced similar results.

### Auxin Quantitations

Auxin quantitations were conducted as previously described (Novák et al., 2012; Jenness et al., 2019). Ions and mass transitions are found in Supplementary Table 2.

### Auxin Transport Assays

Inflorescence transport assays were conducted as described in Kaneda et al. (2011) with some modifications. Briefly, ~15 cm inflorescences were excised at the base and the uppermost floral cluster with supporting stalk and peduncles was removed. All remaining floral organs and peduncles were removed, and the stalks were cut into 2 cm segments. The apical 2 cm and 4–6 cm segments were placed inverted (or upright) in 1:1 ratio of cold IAA: ^3^H-IAA (500 nM each; 22 Ci mmol^−1^, American Radiolabeled Chemicals) in 5 mM MES buffer with 1% sucrose (pH 5.5) for 1 h at room temperature in the dark, then washed and incubated in blank buffer for an additional 12 h. The distal 2 mm were then collected and measured for radioactivity by liquid scintillation counting. For seedling assays, 6% agarose beads (Colloidal Science Solutions, AMB-0601-0010) soaked in a 1:1 ratio of cold IAA: ^3^H-IAA (50 nM each; 24 Ci mmol^−1^, American Radiolabeled Chemicals) were placed over the shoot apex/cotyledonary node or the root–shoot transition zone (RSTZ) of 5.5 days seedlings transferred to a discontinuous filter paper system (Peer and Murphy, 2007). For hypocotyl transport, after incubation 2 mm segments were excised from the RSTZ (10 segments were pooled per replicate). For root transport, 3 mm segments starting from the root tip were collected and pooled separately (10 segments were pooled per replicate). Root tip transport assays were conducted as in Kubeš et al. (2012). Segments were incubated 24 h in 5 ml scintillation cocktail and the amount of radioactivity measured by liquid scintillation counting.

### Histochemical Staining

The 1.650 kb *ABCB11* promoter fragment upstream of the start codon was amplified by PCR then cloned into pENTR/D-TOPO (Thermo Fisher Scientific; primers specified in Supplementary Table 1). The fragment was then transferred into the Gateway compatible vector pGWB3 (Nakagawa et al., 2007) upstream of the *β-glucuronidase* (*GUS*) coding sequence by LR reaction (Thermo Fisher Scientific). Constructs were transformed into Col-0 *via* floral dip (Clough and Bent, 1998). For GUS staining, tissues were incubated in 90% acetone at 4°C for 20 min, then immersed in staining solution (50 mM sodium phosphate buffer, pH 7.0, 0.1% triton X-100, 0.5 mM potassium ferrocyanide, 0.5 mM potassium ferricyanide, and 1 mM X-gluc) and incubated in the dark at 37°C for 5 h, unless otherwise specified. Stained samples were incubated with 70% ethanol at 4°C overnight before imaging. *proABCB11:GUS* inflorescence stems were GUS stained prior to hand sectioning. Cantils were hand sectioned from inflorescence stems and stained using Toluidine Blue O (TBO) prior to imaging a Zeiss Stemi-2000 stereo microscope (Carl Zeiss, Germany) using Lumenera Infinity2 software (Lumenera Corp.).

### Transport Assays in *Schizosaccharomyces pombe*

Yeast assays were conducted using 40 nM ^3^H-IAA (20 Ci mmol^−1^, American Radiolabeled Chemicals) or 50 nM ^3^H-benzoic acid (20 Ci mmol^−1^, American Radiolabeled Chemicals) as previously described (Yang and Murphy, 2009). Assays conducted with ^3^H-benzoic acid alone or 1:1 IAA:benzoic acid (40 nM each) were conducted over 8 min. The ABCB11 expression construct was created by amplifying ABCB11 using primers containing NcoI and XmaI restriction sites (Supplementary Table 1). The digested ABCB11 product was cloned into the corresponding NcoI and XmaI sites of the pREP41 yeast expression vector. Expression vectors were transformed into *S. pombe* by electroporation.

### Protein Sequence Analysis

Protein sequence relationships were inferred by using the Maximum Likelihood method and JTT matrix-based model (Jones et al., 1992). Initial protein alignments were conducted using MUSCLE (Edgar, 2004) and analyses were conducted in MEGA11 (Tamura et al., 2021). The tree with the highest log likelihood (−32,080.72) is shown. Initial tree(s) for the heuristic search were obtained automatically by applying Neighbor-Join and BioNJ algorithms to a matrix of pairwise distances estimated using the JTT model, and then selecting the topology with superior log likelihood value. The tree is drawn to scale, with branch lengths measured in the number of substitutions per site. This analysis involved 21 amino acid sequences. There were a total of 1,447 positions in the final dataset. Structure-based sequence conservation was determined from the MUSCLE sequence alignment and the AlphaFold2 ABCB19 prediction (Jumper et al., 2021; Varadi et al., 2022) using the ConSurf server (Ashkenazy et al., 2016). Colors indicating conserved and flexible residues were applied to the ABCB19 structure by running the ConSurf script in Pymol.

### Statistics

All statistical analyses were performed using JMP PRO 14 (SAS Institute Inc.).

## Results

### Identification of Candidate ABCB Genes

**Figure 1:**
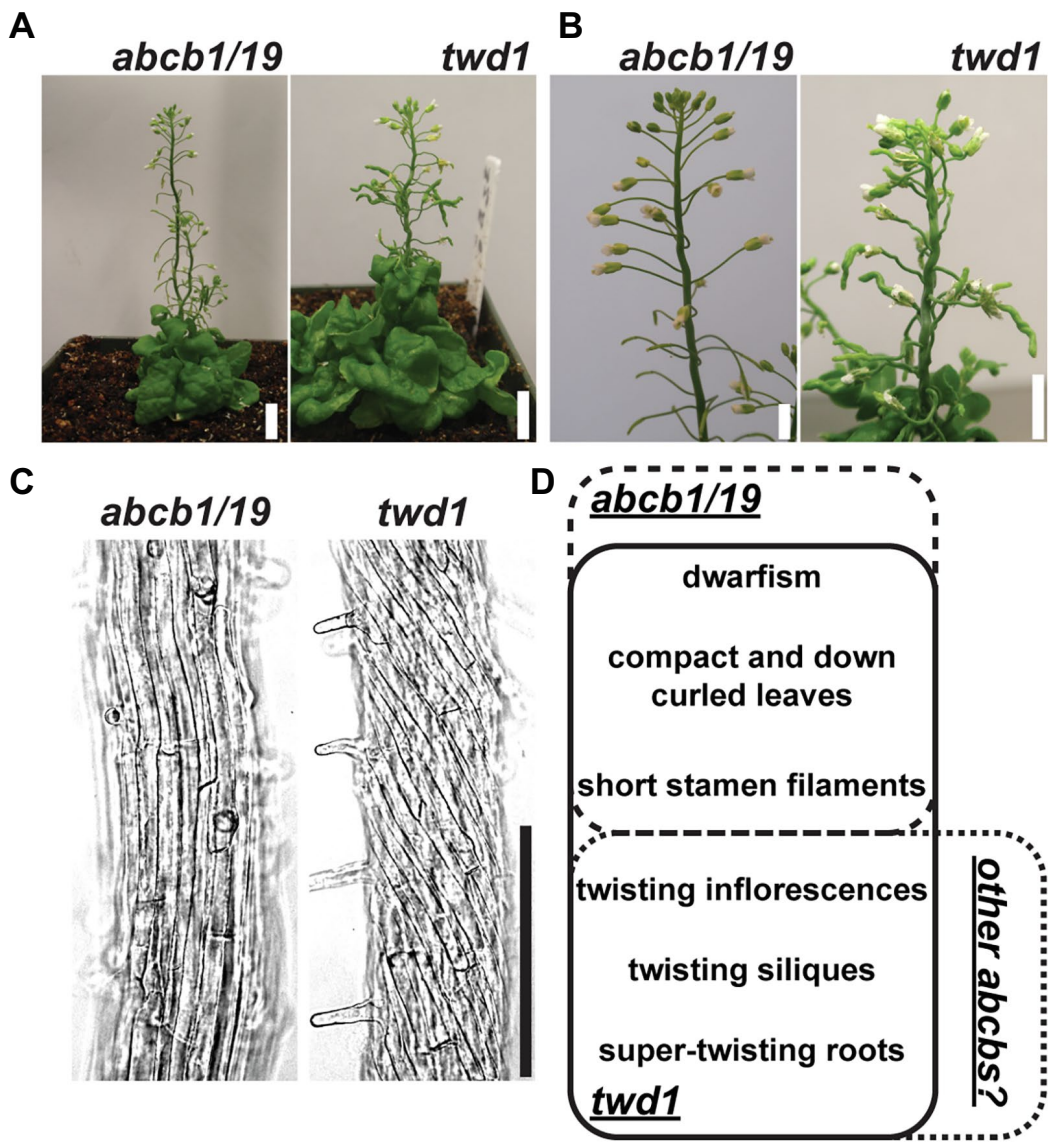
*twd1* exhibits additional root, shoot, and silique twisting not observed in *abcb1/19*. **(A)** Comparison of mature plant phenotypes of *abcb1/19* and *twd1*. **(B)** Close-up of twisting inflorescence stems and siliques observed in *twd1* but not in *abcb1/19*. **(C)** Bright field images *abcb1/19* and *twd1* roots. Images were converted to black and white, and brightness and contrast were adjusted to show cell files. **(D)** Venn diagram of the major phenotypes of *abcb1/19* and *twd1*, and *twd1* phenotypes that may be accounted for in other *abcb* mutants. Scale bars: **(A)** 1 cm; **(B)** 500 μm; **(C)** 200 μm.

The demonstrated dependence of ABCB1 and 19 on TWD1 for proper trafficking and functionality at the PM is apparent in the significant phenotypic overlap between *twd1* and *abcb1/19* double mutants including severe dwarf inflorescence phenotypes and compact and curled rosette and cauline leaves (Figures 1A,B,D; Noh et al., 2001; Geisler et al., 2003; Pérez-Pérez et al., 2004; Blakeslee et al., 2007). *twd1* mutants, however, display additional helical twisting of inflorescence stems and siliques that is not observed in *abcb1/19* double mutants (Figures 1A,B,D; Geisler et al., 2003; Pérez-Pérez et al., 2004). *twd1* mutants also show helical super-twisting roots (Figure 1C; Geisler et al., 2003). This phenotype has been reported both as present and absent in *abcb1/19* mutants which may reflect differences in ecotypic background, mutant alleles, and growth conditions (Geisler et al., 2003; Bouchard et al., 2006; Bailly et al., 2008; Wu et al., 2010; Wang et al., 2013). In the *abcb1/19* double mutant used in this study (*abcb1-100 abcb19-101*) roots exhibit increased waving and turning but not super-twisting cell files (Figure 1C). Additionally, reproducible *twd1*-like cell file twisting was not observed in *abcb1/19* under varying media or sucrose concentrations, light fluencies, or photoperiod (Supplementary Figures 1A–C).

The Arabidopsis genome encodes 21 full-length ABCB isoforms (and the non-transcribed pseudogene ABCB8) that form highly conserved paralogous pairs and sets from gene duplication events (Supplementary Figures 2A–C; Verrier et al., 2008). The auxin transporters *ABCB1*, *4*, and *19* have the highest levels of expression in auxin conducting tissues (Supplementary Figure 3; Klepikova et al., 2016) and have high affinity for TWD1 (Geisler et al., 2003; Wu et al., 2010; Bailly et al., 2013; Wang et al., 2013; Hao et al., 2020). Additionally, previous studies have identified auxin transport contributions from ABCB6 and 20 in shoots, and ABCB21 in roots (Kamimoto et al., 2012; Zhang et al., 2018; Jenness et al., 2019). Although *ABCB2* expression is relatively high, it does not interact with TWD1 (Geisler et al., 2003) and does not transport auxin in yeast (Yang and Murphy, 2009). ABCB11 was previously suggested to contribute to rootward auxin transport in inflorescence stems (Kaneda et al., 2011). However, *ABCB11* is expressed primarily in roots (Supplementary Figure 3) where contribution to auxin transport and *twd1*-like root phenotypes seems more likely. ABCB15-18, and 22 were recently categorized as Auxin Transporting ABCBs (ATAs) based on sequence analysis (Hao et al., 2020). However, based on the moderate expression levels of *TWD1* and stoichiometric requirement for TWD1 activation of more highly expressed ABCBs, the extent to which this cluster of ABCBs requires TWD1 activation will require further experimental evidence. Taken together and considering functional redundancy, these results suggest that the ABCB4/21, 6/20, and 11/12 paralogs are the best candidates to account for the remaining *twd1* twisting phenotypes not observed in *abcb1/19*.

### *abcb4* and *abcb21* Are Not Primary Contributors to the Most Obvious *twd1* Phenotypes

**Figure 2:**
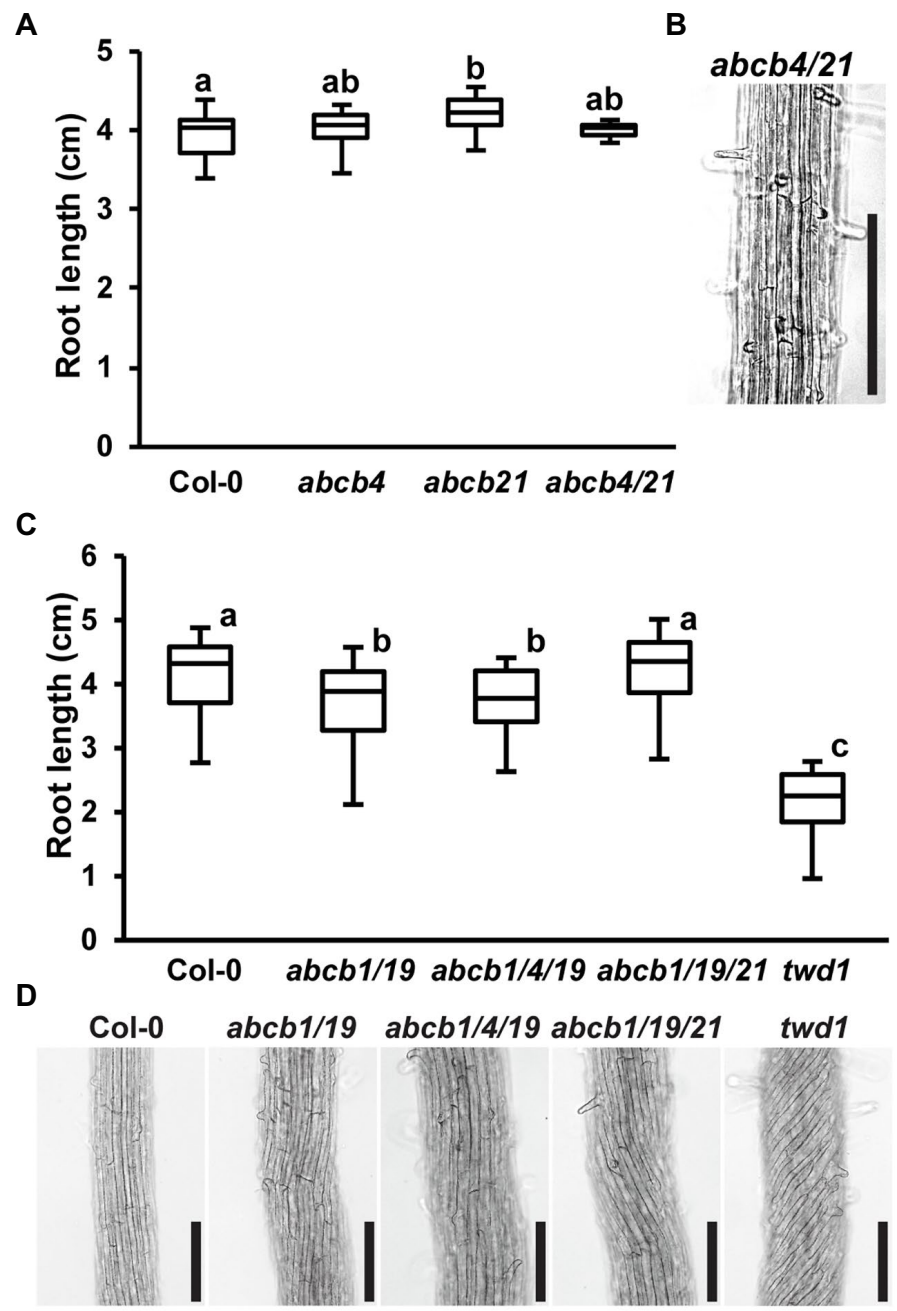
Loss of *abcb4* and *abcb21* does not result in *twd1*-like root phenotypes. **(A)** Root lengths of 7-day light-grown Col-0, *abcb4*, *abcb21*, and *abcb4/21* seedlings. **(B)** Root cell files of 7-day light-grown *abcb4/21* seedlings. **(C)** Root lengths of 7-day light-grown Col-0, *abcb1/19*, *abcb1/4/19*, *abcb1/19/21*, and *twd1* seedlings. **(D)** Root cell files of seedlings in **(C)**. Images in **(B,C)** were converted to black and white, and brightness and contrast were adjusted to show cell files. Lowercase letters indicate statistical difference by pairwise Student’s *t*-test (*p <* 0.05; *n* = 3 replicates of 10–13). Scale bars: 200 μm.

Due to high levels of *ABCB4* expression in the root (Supplementary Figure 3; Klepikova et al., 2016) and interaction with TWD1 (Wu et al., 2010; Hao et al., 2020), it was hypothesized that loss of ABCB4 in addition to ABCB1 and ABCB19 could account for the *twd1* root super-twisting phenotype. *abcb4* mutants share partially overlapping root phenotypes with *twd1* including defects in root hair length and orientation and conditional effects on primary root length (Santelia et al., 2005; Terasaka et al., 2005; Cho et al., 2007; Kubeš et al., 2012). Higher order mutants of *ABCB4* and its paralog *ABCB21* were therefore examined for contribution to *twd1* phenotypes. ABCB4 and ABCB21 do not have compensatory expression in their reciprocal mutant backgrounds, their expression domains do not substantially overlap (Supplementary Figure 3; Santelia et al., 2005; Terasaka et al., 2005; Kamimoto et al., 2012; Kubeš et al., 2012; Klepikova et al., 2016), and no twisting phenotypes were reported in *abcb4/21* aerial tissues (Jenness et al., 2019). Consistent with this, *abcb4/21* did not exhibit any of the highly reproducible *twd1* primary root growth or twisting phenotypes under any growth conditions (Figures 2A,B). It was then hypothesized that additional loss of ABCB4 or ABCB21 in addition to *abcb1/19* could recapitulate the *twd1* root phenotypes. No *twd1*-like root growth or twisting was observed in *abcb1/4/19* or *abcb1/19/21* roots (Figures 2C,D). *abcb1/19/21* mutants were previously reported to exhibit slight alterations in rosette leaf morphology and epidermal cell size compared to *abcb1/19* (Jenness et al., 2019). However, *abcb1*/*4/19* and *abcb1/19/21* triple mutant rosettes and inflorescences were visibly indistinguishable from *abcb1*/*19* and no *twd1*-like twisting phenotypes were observed (Supplementary Figures 4A,B). Together, these results suggested that ABCB4 and ABCB21 are not primary contributors to *twd1* phenotypes and that mis-regulation of other ABCB auxin transporters likely account for the additional root and shoot twisting phenotypes.

### Loss of *ABCB6* and *ABCB20* Accounts for *twd1*-Like Rosette and Inflorescence Phenotypes

**Figure 3:**
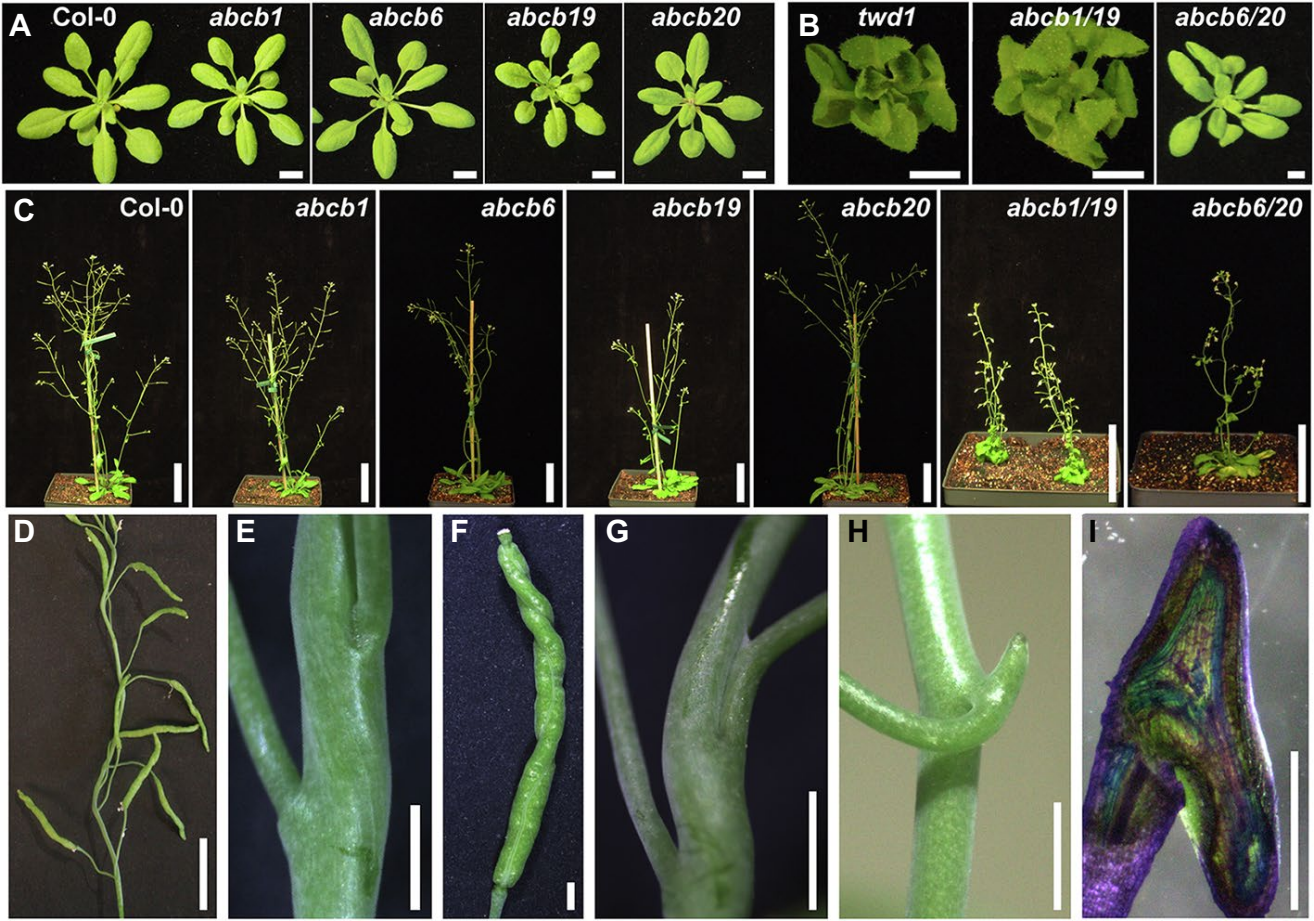
Aerial tissue phenotypes observed in *twd1* can be attributed to loss of ABCB1, 6, 19, and 20 function. **(A)** Single *abcb* mutant rosettes 21 days after germination. **(B)** *twd1* and double *abcb* mutant rosettes 21 days after germination. **(C)** Mature single and double *abcb* mutants 35 days after germination. **(D)** Twisting of inflorescence stems in abc*b6/20* double mutants. **(E,F)** Close-ups images of *abcb6/20* showing **(E)** twisted cell files and **(F)** twisted siliques. **(G)** Occasional stem–pedicel separation defects observed in *abcb6/20* mutants. **(H)** Cantil spur formation in *abcb1/19* grown under 50 μmol m^−2^ s^−1^, 16-h photoperiod. **(I)** Toluidine blue O staining of hand sectioned *abcb1/19* cantil spur from **(H)**. Scale bars: **(A)** 1 cm; **(B,I)** 0.5 cm; **(C)** 5 cm; **(D,H)** 1 cm; **(E–G)** 1 mm.

*ABCB6* and *ABCB20* are the most highly expressed ABCB auxin transporters in aerial tissues after *ABCB1* and *19* (Supplementary Figure 3; Klepikova et al., 2016). Single and double mutants of these transporters were therefore analyzed for defects in leaf, stem, and silique morphology. At the pre-flowering stage, both *abcb1/19* and *twd1* exhibited compact rosettes with severe downward leaf curling (Figures 3A,B; Noh et al., 2001; Geisler et al., 2003; Pérez-Pérez et al., 2004; Blakeslee et al., 2007). These phenotypes can be attributed primarily to loss of ABCB19 function as it is the only single *abcb* mutant to show substantial defects in leaf morphology reported to date (Figure 3A; Supplementary Figure 5; Noh et al., 2001; Zhao et al., 2013; Jenness et al., 2020). Single *abcb1*, *abcb6*, and *abcb20* mutants did not exhibit morphological leaf defects (Figure 3A; Supplementary Figure 5) in part due to compensatory expression and redundant function between paralogous transporters as previously described (Zhang et al., 2018; Jenness et al., 2019). Double *abcb6/20* rosettes were compact, tended to laterally twist and skew, and had increased leaf down curling and wrinkling which resembled *abcb19* (Figure 3B; Supplementary Figure 5). These results point to the loss of ABCB1 and ABCB19 as the primary contributors to *twd1* leaf phenotypes with additional contributions from ABCB6 and ABCB20.

**Figure 4:**
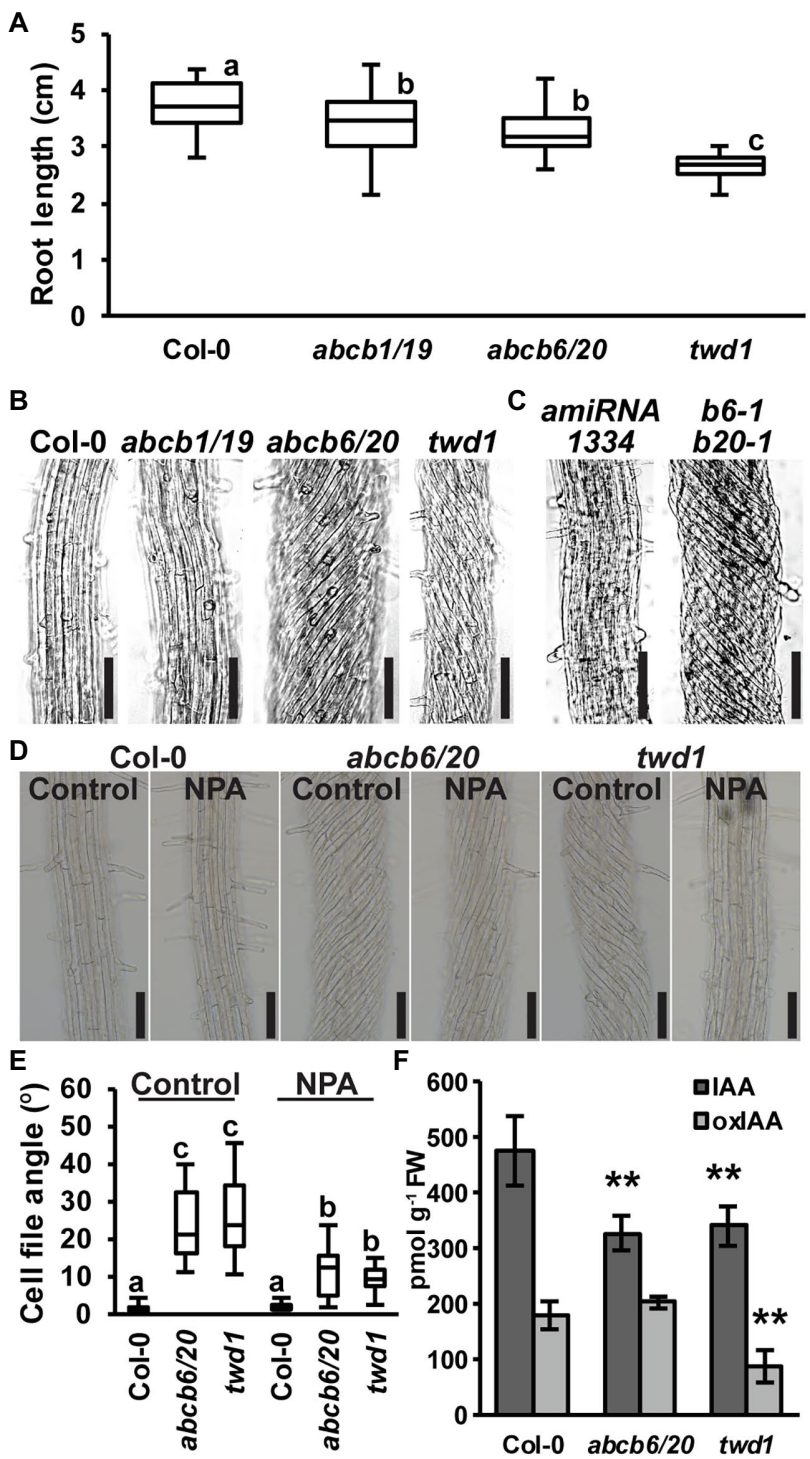
Root phenotypes observed in *twd1* can be attributed to loss of ABCB1/19 and 6/20 function. **(A)** 7-day primary root lengths (*n* = 3 replicates of 10–13). **(B,C)** Bright field images of the distal elongation and maturation zone in 7-day seedling roots. Images in **(B,C)** were converted to black and white, and brightness and contrast were adjusted equally within each set to show cell files. **(D)** Treatment with 1 μM NPA suppressed root twisting in *abcb6/20* and *twd1*. Brightness and contrast were adjusted equally to show cell files. **(E)** Quantification of cell file angles in **(D)**. Data shown are means ± SE (*n* = 3 replicates of 10–11). **(F)** Quantification of IAA and oxIAA in 5.5-day light-grown roots. Data shown are means ± SD (*n* = 4–5 independent pools of 25 mg tissue). Lowercase letters indicate statistical difference by all pairwise Student’s *t*-test (*p <* 0.05). Asterisks indicate statistical differences by Student’s *t*-test (^**^p < 0.01). Scale bars: 100 μm.

After flowering, *abcb1/19* and *twd1* developed short inflorescence stems (Figure 1A; Noh et al., 2001; Geisler et al., 2003; Pérez-Pérez et al., 2004; Blakeslee et al., 2007). Consistent with previous reports, *abcb1* and *abcb19* single mutants exhibited slightly decreased inflorescence lengths, which were exaggerated in *abcb1/19* (Figure 3C; Table 1; Noh et al., 2001; Blakeslee et al., 2007). While *abcb6* and *abcb20* inflorescences were not different and slightly longer than Col-0, respectively, *abcb6/20* double mutants were severely dwarfed (Figure 3C; Table 1). Single and double *abcb1* and *abcb19* mutants had reduced cauline leaf branches (Table 1). Examination of internodes in *abcb1/19* and *abcb6/20* revealed that both double mutants had drastically reduced internode lengths (Table 1). Additionally, *abcb6/20* mutants had fewer secondary inflorescences (Table 1). Comparison of *twd1* inflorescence phenotypes was not possible due to the severe delay in bolting.

**Table 1:** Inflorescence measurements of *abcb1*, *19*, *6*, and *20* single and double mutants.

	Inflorescence length (cm)	Cauline leaf branches	Bottom internode length (cm)	Secondary inflorescences
Col-0	36.4 ± 4.0	3.7 ± 0.5	5.5 ± 0.6	2.5 ± 0.7
*abcb1*	31.2 ± 2.7^*^	2.4 ± 0.5^*^	3.1 ± 0.8*	3.2 ± 0.4
*abcb19*	27.8 ± 2.1^*^	1.8 ± 0.4^*^	4.6 ± 0.3	3.0 ± 0.7
*abcb1/19*	12.2 ± 1.4^*^	2.0 ± 0.5^*^	0.6 ± 0.3^*^	2.1 ± 0.6
*abcb6*	36.1 ± 4.0	4.1 ± 0.7	5.2 ± 1.7	2.3 ± 0.8
*abcb20*	41.0 ± 2.6^*^	3.1 ± 0.6	4.7 ± 1.2	2.9 ± 0.6
*abcb6/20*	14.0 ± 3.1^*^	3.6 ± 1.3	1.6 ± 1.2^*^	1.5 ± 1.1^*^

Plants were grown in soil for 5 weeks under 100 μmol m^−2^ s^−1^ white light, 16 h photoperiod. Data shown are means ± SD (*n* = 10 mature plants). Asterisks indicate statistical difference from Col-0 by Student’s *t*-test (*p* < 0.05).

*abcb1/19* exhibited increased stem and silique waving and skewing but *twd1*-like helical twisting of these organs was not observed (Figures 1A,B). Instead, these twisting phenotypes could be attributed to loss of ABCB6 and ABCB20 function (Figures 3D–F). While not observed in the single mutants, *abcb6/20* exhibited twisted inflorescence stems (Figures 3D,E), twisted siliques (Figure 3F), and lateral organ separation defects (Figure 3G) which resemble the fused branches and pedicels observed in *abcb19* (Zhao et al., 2013). Long-day-grown *abcb6/20* and *abcb1/19* mutants also developed expanded pedicel–stem junctions that resembled the recently described cantil cuffs observed in Col-0 under short-day conditions (Figure 3E; Supplementary Figures 6A–C; Gookin and Assmann, 2021). These phenotypes were exaggerated in *abcb1/19* under low light (Figure 3H) forming cantil spurs that were accompanied by ectopic vasculature formation (Figure 3I).

Overall, the leaf and inflorescence phenotypes associated with loss of ABCB6 and 20 are consistent with those previously reported (Zhang et al., 2018). Together these results point to the loss of ABCB6 and 20 as the primary contributors to the aerial organ twisting observed in *twd1*, and the combinatorial effects of loss of ABCB1, 19, 6, and 20 function in the severe compact leaf and dwarf inflorescence phenotypes. It was hypothesized that an *abcb1/19/6/20* quadruple mutant would completely recapitulate the *twd1* phenotype but the isolation of *abcb1/19/6/20*, *abcb1/19/6*, and *abcb1/6* mutants was not successful by crossing due to the close proximity of *ABCB1* and *ABCB6* on chromosome 2. Analysis of *abcb1/19/20* did not reveal any visible differences in rosettes or inflorescence from *abcb1/19* (Supplementary Figures 7A,B).

### Loss of *ABCB6* and *ABCB20* Accounts for *twd1*-Like Helical Root Twisting

Previous analyses found that *abcb6*/*20* mutants exhibit minor root elongation defects, but root twisting phenotypes were not reported (Zhang et al., 2018). Consistent with these results, measurement of *abcb6*/*20* double mutants revealed slight reductions in primary root elongation similar to *abcb1*/*19* but not as severe as *twd1* (Figure 4A). However, examination of cell files in the distal elongation and maturation zones also revealed extreme twisting of the epidermis in *abcb6/20* that resembled *twd1* that was not present in *abcb1/19* (Figure 4B). To further validate the root twisting phenotype, the previously characterized *amiRNA1334 ABCB6/20* knock-down and *abcb6-1 abcb20-1* double mutant lines were also examined (Zhang et al., 2018). While *amiRNA1334* showed increased cell file irregularity which was consistent with reduced *ABCB6* and *ABCB20* expression, the *abcb6-1 abcb20-1* double mutant displayed the same non-handed cell file twisting as *abcb6/20* (Figure 4C). Further loss of *abcb20* in addition to *abcb1/19* (*abcb1/19/20*) enhanced root growth compared to *abcb1/19* (Supplementary Figures 7C,D) but no cell file twisting was observed. These results demonstrate that cell file twisting only occurs with loss of both *abcb6* and *abcb20*, which is consistent with functional redundancy between these paralogs as described (Zhang et al., 2018). Previous reports have shown that exogenous treatment with NPA inhibits cellular auxin efflux in part by disrupting TWD1 interactions with ABCB auxin transporters (Bailly et al., 2008; Christie et al., 2011) and NPA can suppress cell file twisting in *twd1* roots (Wu et al., 2010; Wang et al., 2013). Treatment with 1 μM NPA suppressed cell file twisting in *abcb6*/*20* to angles that were not significantly different from NPA-treated *twd1* (Figures 4D,E). Some *abcb6/20* roots showed some residual twisting that may reflect minor contributions from ABCB1/19. Quantification of free IAA levels in roots revealed both *abcb6/20* and *twd1* have significantly reduced auxin levels (Figure 4F). Additionally, *twd1* also contained reduced levels of the auxin oxidation catabolite oxindole-3-acetic acid (oxIAA; Figure 4F) suggesting additional auxin homeostasis mechanisms are differentially activated in *twd1* and *abcb6/20*. These results suggest the combined loss of ABCB1, 19, 6, and 20 function contributes to the root elongation defects observed in *twd1*, and that *abcb6* and *20* are the primary contributors to the NPA-sensitive root cell file twisting phenotype.

### ABCB11 Does Not Contribute to Long-Distance Auxin Transport Streams

**Figure 5:**
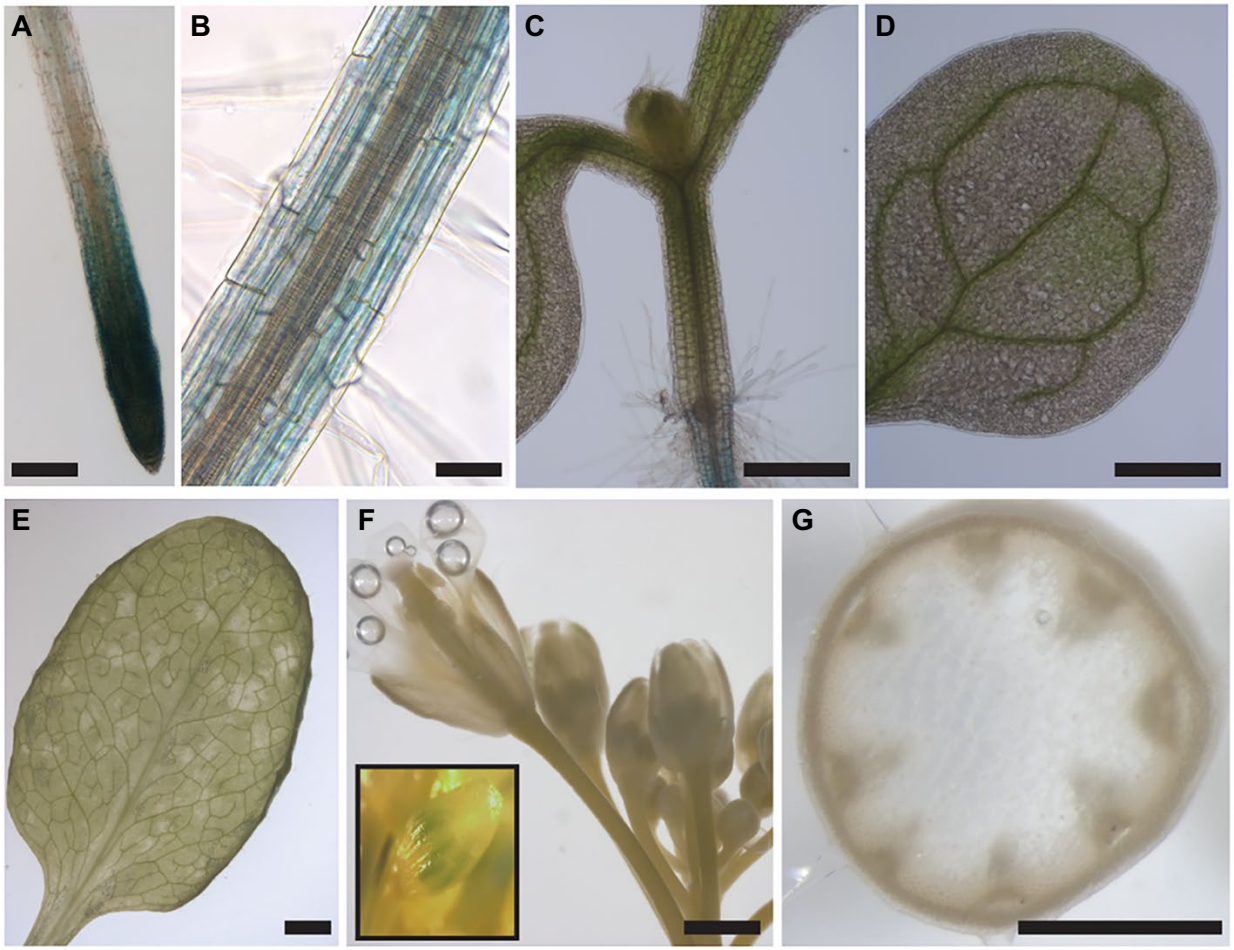
*ABCB11* is primarily expressed in the root and root tip. **(A)** Strong *proABCB11:GUS* expression is observed in the root tip. Roots were stained for 1 h. **(B)** Moderate *proABCB11:GUS* expression is observed in the epidermis, cortex, and endodermis of the mature root. **(C–G)** *proABCB11:GUS* expression is not observed in the **(C)** hypocotyl, shoot apex, or petioles, **(D)** cotyledons, **(E)** rosette leaves, **(F)** apical regions of the inflorescence stem or mature flowers, or **(G)** upper 3–4 cm of the inflorescence stem. Occasional staining could be observed in young unopened flowers (**E**, inset). Stems in **(G)** were GUS stained prior to hand sectioning. Scale bars: **(A,F,G)** 500 μm, **(B,F)** 1 mm, **(C,E)** 2 mm, **(D)** 100 μm.

Although combined loss of *abcb1/19* and *abcb6/20* account for all the major *twd1* phenotypes, *ABCB11* has also been implicated in auxin transport in inflorescence stems (Kaneda et al., 2011) and is the next highest *ABCB* transporter expressed in the root after *ABCB19*, *4*, and *1* (Supplementary Figure 3). Tissue-specific expression was confirmed by histochemical analysis of a 2.104 kb *ABCB11* promoter fragment fused to *β-Glucuronidase* (GUS) in Col-0. Strong GUS staining was observed in the root tip and moderate expression observed in the mature root outside of the vasculature (Figures 5A,B). No staining was detected in the hypocotyl, shoot apex, cotyledons, rosette leaves, or apical regions of the inflorescence stem including mature flowers (Figures 5C–F). Occasional staining was observed in young, unopened flowers (Figure 5F). Previously, strong *ABCB11* expression associated with the vasculature was reported in the upper 3 cm of the inflorescence stem (Kaneda et al., 2011). However, no observable GUS staining could be detected in this region with the *ABCB11* promoter construct used in this study (Figure 5G). The results presented here are more consistent with the expression patterns found in RNA-seq and microarray databases (Zimmermann, 2004; Klepikova et al., 2016).

Previous reports suggested that ABCB11 contributes to rootward auxin transport in inflorescence stems (Kaneda et al., 2011). However, according to the Arabidopsis Biological Resource Center (ABRC) the T-DNA insertion line used in those studies (SALK_094249), understandably misreported as an insertion in *ABCB11*, instead contains an insertion in the adjacent, paralogous, and highly similar gene *ABCB12*. Therefore, two new alleles of *abcb11* (SALK_057628 and SALK_037942), designated *abcb11-1* and *abcb11-2*, were isolated and confirmed to have transcript levels below the limit of detection (Supplementary Figures 8A,B). Additionally, no change in *ABCB12* expression was observed in *abcb11-1* suggesting *ABCB12* does not compensate for loss of *ABCB11* function (Supplementary Figure 8C). Based on the lack of compensation and extremely low *ABCB12* expression in all tissues (Supplementary Figure 3), *ABCB12* was not examined any further.

**Figure 6:**
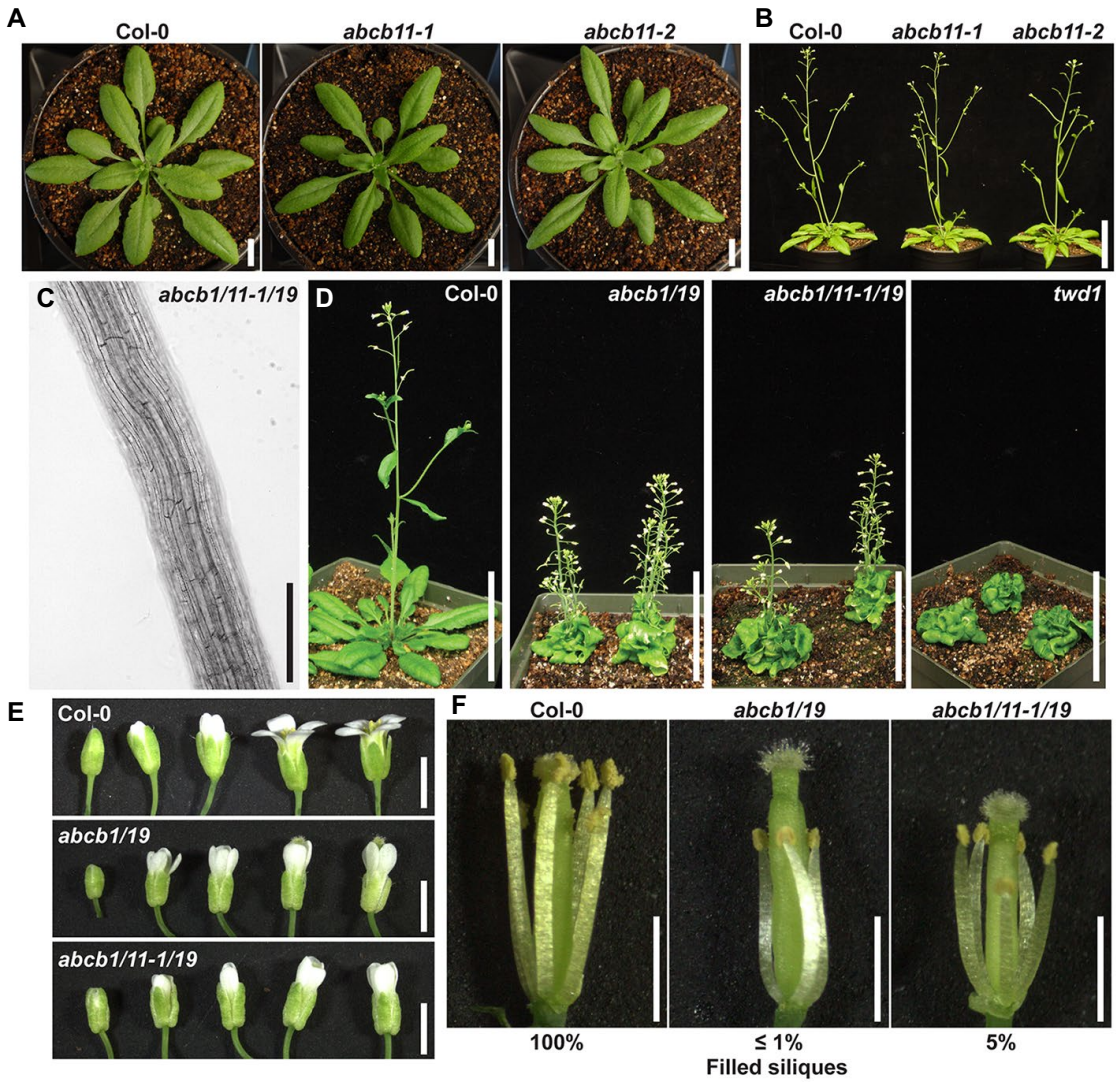
*ABCB11* contributes to auxin phenotypes supplementary to *ABCB1/19*. **(A)** Col-0 and *abcb11* rosettes at time of bolting. **(B)** Col-0 and *abcb11* mature plants. **(C)** Root cell files of 7-day light-grown *abcb1/11-1/19* seedlings. Images were converted to black and white, and brightness and contrast were adjusted to show cell files. **(D)** 35-day rosettes and inflorescences of Col-0, *abcb1/19*, *abcb1/11-1/19*, and *twd1*. **(E)** Flower series of Col-0, *abcb1/19*, and *abcb1/11-1/19*. **(F)** *Col-0*, *abcb1/19*, and *abcb1/11-1/19* flowers with petals removed. Scale bars: **(A)** 1 cm, **(B,C)** 5 cm, **(D)** 200 μm, **(E,F)** 1 mm.

Phenotypic analysis of *abcb11-1* and *abcb11-2* mutants revealed slight defects in primary root elongation, but no other phenotypic differences from Col-0 were observed under stable conditions (Figures 6A,B; Table 2). However, *abcb11-1* seedlings grown on vertical plates did exhibit increased incidence of root waving and skewing in response to mechanical perturbation or directional light. Root waving and skewing phenotypes are primarily regulated by auxin transport in the root tip (Okada and Shimura, 1990; Mullen et al., 1998; Hu et al., 2021) where *ABCB11* is primarily expressed and can be quantified in seedlings grown on vertical media set to varying inclination angles (Okada and Shimura, 1990; Oliva and Dunand, 2007). To quantify the contribution of ABCB11 to root waving seedlings were grown on media set at varying inclination angles. When plates were set to 15 degrees from vertical, *abcb11-1* showed increased root waving compared to Col-0, but no significant difference in primary root length (Supplementary Figures 9A–C).

**Table 2:** Phenotypic analysis of *abcb11* mutants.

**Seedling phenotypes**				
	**5 days primary root length (cm)**	**7 days primary root length (cm)**	**10 days primary root length (cm)**	**10 days LR density (LR cm^−1^)**
Col-0	0.97 ± 0.09	1.99 ± 0.28	4.23 ± 0.49	0.41 ± 0.04
*abcb11-1*	0.85 ± 0.01^*^	1.72 ± 0.33^*^	3.74 ± 0.41^*^	0.40 ± 0.09
*abcb11-2*	0.77 ± 0.2^*^	1.55 ± 0.21^*^	3.53 ± 0.32^*^	0.46 ± 0.02
**Mature phenotypes**				
	**Inflorescence length (cm)**	**Cauline leaf branches**	**Bottom internode length (cm)**	**Secondary inflorescences**
Col-0	23.5 ± 3.1	3.7 ± 0.5	4.62 ± 1.4	2.7 ± 0.7
*abcb11-1*	22.0 ± 3.4	3.5 ± 0.5	4.4 ± 0.9	2.1 ± 0.7
*abcb11-2*	20.9 ± 3.2	3.5 ± 0.6	5.0 ± 1.1	2.3 ± 0.6

Seedlings were grown on ¼ MS, 1 g L^−1^ MES, 0.5% sucrose, 0.8% agar (pH 5.6) under 100 μmol m^−2^ s^−1^ continuous white light. Mature plants were grown in soil for 4 weeks under 100 μmol m^−2^ s^−1^ white light, 16 h photoperiod. Data shown are means ± SD (*n* = 25–30 seedlings or 10 mature plants). Asterisks indicate statistical differences from Col-0 by Student’s *t*-test (*p* < 0.05).

Further analysis showed *abcb1/11/19* triple mutants, generated using *abcb11-1*, phenotypically resembled *abcb1/19* and did not exhibit any root, inflorescence, or silique twisting (Figures 6C,D). In aerial tissues, a slight increase in silique filling from self-pollination was observed in *abcb1/11/19* compared to *abcb1/19*. Decreased auxin transport in *abcb1/19* anthers results in short anther filament length and reduced self-pollination (Figures 6E,F; Noh et al., 2001; Cecchetti et al., 2008; Titapiwatanakun et al., 2009). In addition to the short anther phenotype associated with *abcb1/19*, *abcb1/11/19* also exhibited shorter pistils that allowed for a small amount of self-pollination (Figures 6E,F). This resulted in ~5% filled siliques by the time flowering was complete compared to 100% in Col-0 and ≤1% in *abcb1/19*. This suggests ABCB11 regulates auxin transport required for pistil elongation.

**Figure 7:**
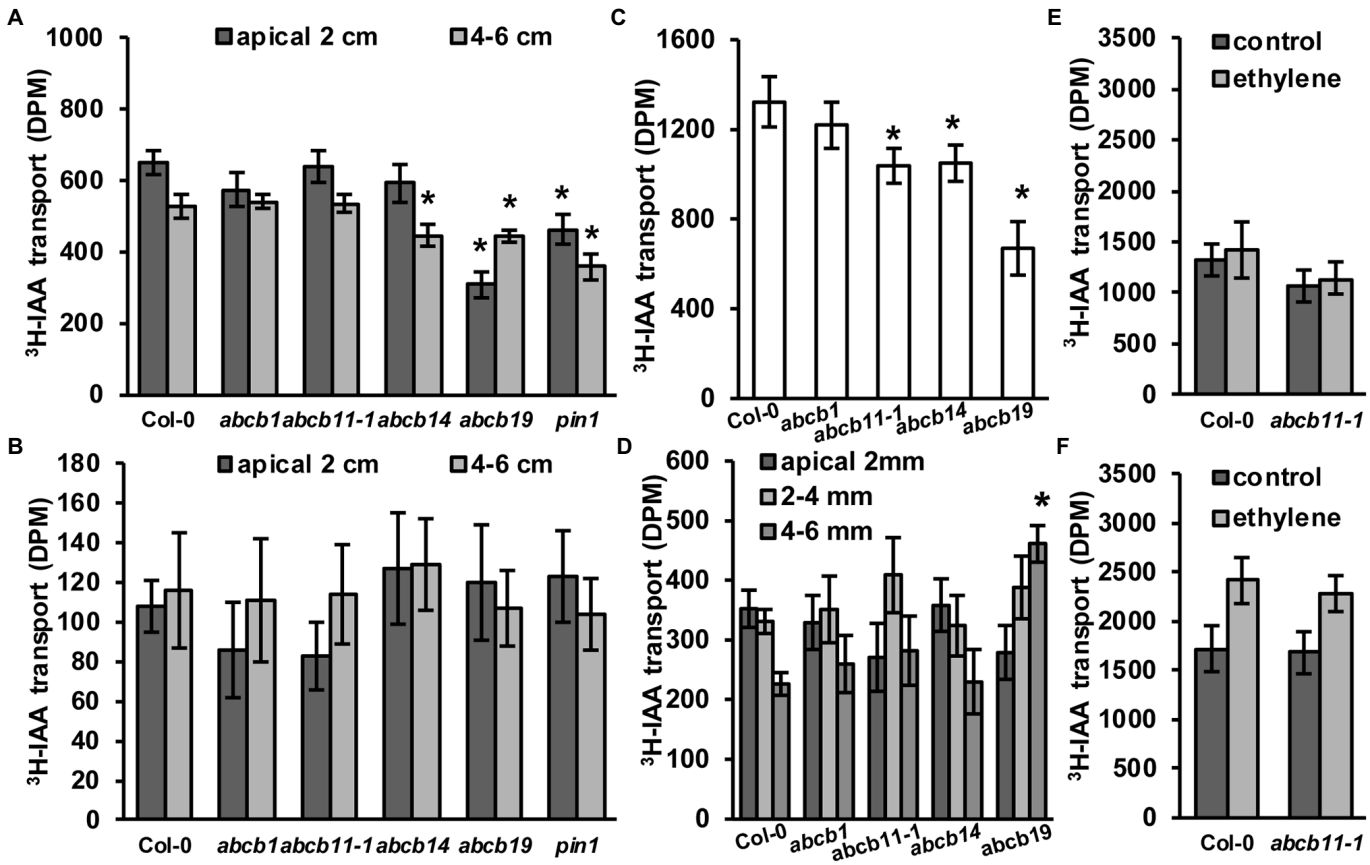
*abcb11-1* mutants exhibit minor defects in root auxin transport. **(A,B)** ^3^H-IAA transport in **(A)** upright (rootward/basipetal) and **(B)** inverted Col-0, *abcb1*, *abcb11*, *abcb14*, *abcb19*, and *pin1* inflorescence stems segments. Assays were conducted as in Kaneda et al. (2011) with modifications as noted in the Materials and Methods section. Data shown are means ± SD (*n* = 3 pools independent of five segments). **(C,D)** ^3^H-IAA transport in Col-0, *abcb11*, *abcb14*, and *abcb19* seedlings from the shoot apex to **(C)** the root–shoot transition zone (RSTZ) or **(D)** the root tip. Data shown are means ± SD (*n* = 3 independent pools of 10). **(E,F)** Shootward auxin transport from the root tip with ^3^H-IAA placed on the **(E)** columella or **(F)** quiescent center. Shootward assays were conducted as in Kubeš et al. (2012). Data shown are means ± SD (*n* = 3 independent pools of 10). Asterisks indicate statistical difference from Col-0 by Student’s *t*-test (^*^p < 0.05).

To see if *abcb11-1* exhibited defects in rootward auxin transport, inflorescence transport assays were conducted using *abcb11-1* according to Kaneda et al. (2011) with some modifications as defined in the Materials and Methods section. Col-0, *abcb1*, *abcb14*, *abcb19*, and *pin1* were included as controls. In the apical 2 cm segments of *abcb19* and *pin1* auxin transport was reduced by ~52% and ~28%, respectively (Figure 7A). In the 4–6 cm segments, transport was reduced in *abcb19* and *pin1* by ~15% and ~32% (Figure 7A). A slight reduction in transport was observed in *abcb14* lower segments (Figure 7A), which is consistent with previous reports (Kaneda et al., 2011). *abcb11-1* mutants did not have any differences in auxin transport with either stem segment. No differences were observed when stems were placed upright in the ^3^H-IAA solution (Figure 7B). These results are consistent with the lack of *ABCB11* expression in inflorescence stems (Figure 5G; Supplementary Figure 3; Zimmermann, 2004; Klepikova et al., 2016) and suggest ABCB11 does not participate in auxin transport in these tissues. This function can instead be attributed to ABCB12 as previously described (Kaneda et al., 2011).

Rootward auxin transport in *abcb11-1* seedlings was also analyzed (Figure 7C). The greatest reduction (~43%) was observed in *abcb19*, while transport in *abcb1* and *abcb11-1* was reduced ~16% and ~17%, respectively. Auxin transport from the shoot apex to the root–shoot transition zone was significantly reduced in *abcb11*, *abcb14*, and *abcb19* (Figure 7C). Since ABCB14 was initially identified as a malate/citrate transporter (Lee et al., 2008), auxin transport was also assessed when malate was co-applied to the seedling shoot apex. Competition with a 5:1 molar ratio of malate:^3^H-IAA reduced auxin transport ~24% in Col-0 (Supplementary Figure 10). Together these results suggested competitive inhibition of other auxin transport components in these respective mutants contribute to rootward auxin flux. Conversely, *abcb14* exhibited close to zero reduction. This suggests that competition of ^3^H-IAA with malate reduces ABCB14 mediated auxin transport. Transport assays with radiolabeled malate were not feasible due to rapid metabolism of applied malate.

As *ABCB11* is moderately expressed in the seedling root (Figures 5A,B; Supplementary Figure 3) and slight defects are observed in *abcb11-1* and *abcb11-2* root growth (Table 2), auxin transport from the RSTZ to the root apex was assayed. Transport in *abcb1* and *abcb19* was assayed as a positive control, and in *abcb14*, which is not expressed in the root, as a negative control. Only *abcb19* exhibited a reduction in auxin transport to the root apex, but *abcb11-1* exhibited a slightly increased accumulation at 2–4 mm from the apex (Figure 7D). These alterations in hypocotyl and root transport in *abcb11-1* mutants were reminiscent of the reduced transport in *abcb4* hypocotyls due to the reduced auxin sink in the root (Terasaka et al., 2005). This suggested ABCB11 may have some limited function in auxin transport at the root tip where it is primarily expressed (Figure 5A; Supplementary Figure 3). However, shootward transport of auxin from the columella or cells surrounding the quiescent center was not different from Col-0 (Figures 7E,F). Shootward auxin transport from these sites in *abcb11-1* in the presence of ethylene was also found to be non-different from Col-0, similar to what was observed *in abcb4* (Kubeš et al., 2012).

**Figure 8:**
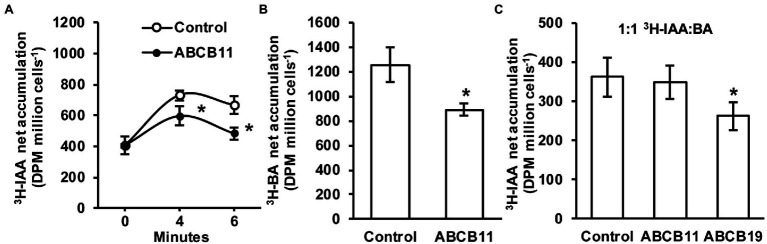
Transport activity of ABCB11 expressed in *Schizosaccharomyces pombe*. **(A)** ^3^H-IAA accumulation in *S. pombe* expressing ABCB11 or empty vector control plasmid. Data shown are means ± SD (*n* = 4 independent transformations). **(B)** ^3^H-benzoic acid (BA) accumulation in *S. pombe* expressing ABCB11 or empty vector control plasmid. Data shown are means ± SD (*n* = 4 independent transformations). **(C)** Competition of ^3^H-IAA with cold benzoic acid (BA; 1:1 molar ratio) in *S. pombe* expressing ABCB11, ABCB19, or empty vector control plasmid. Data shown are means ± SD (*n* = 4 independent transformations). Asterisks indicate statistical difference by Student’s *t*-test (^*^p < 0.05).

Since no clear role for ABCB11-mediated auxin transport was found *in planta* it was hypothesized that ABCB11 may transport alternate substrates. To test this, ABCB11 was analyzed for auxin transport activity when expressed in *S. pombe*. Cells expressing ABCB11 accumulated ~18% less ^3^H-IAA after 4 min and ~27% less after 6 min compared to empty vector controls (Figure 8A). However, expression of ABCB11 also reduced ^3^H-benzoic acid accumulation (Figure 8B). Competition of ^3^H-IAA with benzoic acid (1:1 molar ratio) reduced ^3^H-IAA transport to control levels in cells expressing ABCB11 but not ABCB19 (Figure 8C). This is consistent with previous reports showing benzoic acid is not a substrate for ABCB19 (Yang and Murphy, 2009). These results, along with the lack of auxin related phenotypes and lack of consistent auxin transport defects *in planta*, suggest that ABCB11 has the capacity to transport auxin but likely transports alternate substrates *in vivo*.

## Discussion

The *twisted dwarf 1* (*twd1*) and double *abcb1/19* mutants exhibit significant overlap in morphology including reduced primary root elongation, severely compact and curled leaves, and dwarfed inflorescence height due to reduced long-distance auxin transport (Geisler et al., 2017). However, *twd1* also exhibits twisting roots, inflorescence stems, and siliques that are not observed in *abcb1/19*. The results presented here suggest that all major *twd1* phenotypes are accounted for with the loss of ABCB1, 6, 19, and 20 function and that loss of other well-documented TWD1 interactions with other cellular components provide minor contributions to morphological phenotypes.

Several reports have consistently found that *abcb19* and *abcb1*/*19* mutants exhibit ≥50% reductions in polar auxin transport that result in decreased root length, decreased inflorescence height, short anther filaments, and altered leaf morphology (Noh et al., 2001; Geisler et al., 2003, 2005; Blakeslee et al., 2007; Wu et al., 2007; Christie et al., 2011; Sukumar et al., 2013; Zhao et al., 2013; Bennett et al., 2016; Jenness et al., 2019, 2020). The lack of phenotypes associated with *abcb1* single mutants is attributed to compensatory expression of *ABCB19* (Blakeslee et al., 2007; Jenness et al., 2019). Although organ separation defects have been described in an *abcb19-5* allele (Zhao et al., 2013) that are not observed in other *abcb19* null mutants, inflorescence and silique twisting has not been reported in *abcb1*, *abcb19*, or *abcb1/19*.

The results presented here suggest the combined loss of *ABCB1/19* and *ABCB6/20* contribute to the severe dwarf and twisting rosette and inflorescence phenotypes associated with *twd1*. Rosettes of *abcb1/19* were almost indistinguishable from *twd1* suggesting ABCB1 and ABCB19 have a primary function in rosette leaf development. *abcb6/20* rosettes were also compact, although not to the same extent as *abcb1/19* or *twd1*. While *abcb1/19* leaves were more downward curled, *abcb6/20* tended to curl and skew laterally. Similar trends were observed in inflorescence stems, where loss of *ABCB1/19* and *ABCB6/20* was associated with decreased inflorescence height, but helical stem, and silique twisting was only observed in *abcb6/20*.

In the root, helical twisting has been reported as present (Geisler et al., 2003; Bouchard et al., 2006; Bailly et al., 2008) and missing (Wu et al., 2010) in *abcb1/19*. These differences have been attributed to differences in alleles and ecotypic backgrounds (Wang et al., 2013). The *abcb1/19* double mutants used in this study displayed reduced root elongation with increased root waving and irregular growth, but epidermal cell file twisting was never observed. Instead, *abcb6/20* mutants displayed helical non-handed cell file twisting. Treatment with NPA suppressed twisting to a similar extent in *abcb6/20* and *twd1*, and was consistent with previous reports with *twd1* (Wu et al., 2010; Wang et al., 2013). Although a subset of ABCBs and TWD1 bind NPA directly (Murphy et al., 2002; Bailly et al., 2008; Kim et al., 2010), NPA rescues twisting in the absence of TWD1 and ABCB6/20 suggesting the rescue is independent of TWD1 and ACTIN-dependent ABCB trafficking. Although the exact mechanism is not known it is likely that ABCB6/20 or TWD1 loss of function leads to helical twisting *via* ectopic auxin accumulations, differential cell expansion, and geometric constraints as has been described for other twisting mutants (Weizbauer et al., 2011). This twisted growth likely also involves PIN upregulation and PIN-directed transport as overexpression of PINs leads to hypocotyl twisting that can be rescued by NPA treatment (Mravec et al., 2008).

Attempts were made to generate an *abcb1/19/6/20* quadruple mutant as it would be expected to fully resemble *twd1*. However, no combinations of *abcb1/6* were isolated presumably due to infrequent recombination between *ABCB1* and *ABCB6*, which are separated by only 983,313 nucleotides on chromosome 2. Analysis of *abcb1/19/20* mutants did not reveal any additional phenotypes to *abcb1/19* double mutants, which is consistent with the functional redundancy of ABCB6 and 20 reported here and previously (Zhang et al., 2018). Generation of the *abcb1/19/6/20* quadruple mutant will likely require use of a CRISPR-CasX approach of extraordinary finesse, as attempts to generate such mutants so far have inactivated a larger number of *ABCB* genes.

Phenotypic characterizations provide little insight into ABCB11 function. *ABCB11* expression is primarily limited to the root, however, and no major differences in auxin transport in the root were observed in *abcb11* mutants. In flowers, combined loss of ABCB1, 11, and 19 contributed to reduce pistil elongation, suggesting ABCB11 may contribute to auxin transport supplementary to ABCB1 and 19 in this tissue. *ABCB11* is also expressed in anthers (Klepikova et al., 2016), however, loss of ABCB11 did not enhance the filament elongation defects associated with *abcb1/19*, again pointing to a supplementary role in auxin transport. These results support a role for TWD1 in regulating auxin-dependent floral organ elongation (Pérez-Pérez et al., 2004) *via* ABCB1, 11, and 19. Additional roles for TWD1 in regulating BRI1 and brassinosteroid-dependent pollen development are likely (Vogler et al., 2014; Zhao et al., 2016). When expressed in *S. pombe*, ABCB11 does increase auxin export, but this activity was competitively eliminated by benzoic acid with relative ease. This suggests ABCB11 may function in transport of additional amphipathic or aromatic organic acid substrates *in planta*. Less specific auxin transport activity is observed with the malate/citrate transporter ABCB14 (Lee et al., 2008), which appears to mediate transport of exogenously applied auxin (Kaneda et al., 2011), but exhibits preferential transport of simple organic acids (Supplementary Figure 10; Lee et al., 2008; Yang and Murphy, 2009). Since endogenous malate/citrate levels are several fold higher than auxin, ABCB14 contribution to auxin transport is expected to be minimal.

The exact mechanism by which such a small amount of TWD1 maintains the trafficking and activity of this relatively abundant group of ABCB transporters is still unclear, but could involve its role in mediating actin bundling (Zhu et al., 2016). Emerging methods including single molecule interaction visualizations and new tools to detect ABC transporter function in real time may be able to provide further insight in the future.

## Data Availability Statement

The raw data supporting the conclusions of this article will be made available by the authors, without undue reservation.

## Author Contributions

MJ and AM planned and designed the research and wrote the manuscript with input from all authors. MJ, RT, GB, WT, YZ, CP, and AM performed the experiments. MJ, RT, WT, and AM analyzed the data. All authors contributed to the article and approved the submitted version.

## Funding

This work was supported by the Department of Energy, Basic Energy Sciences, grant no. DE-FG02-06ER15804 to AM.

## Conflict of Interest

The authors declare that the research was conducted in the absence of any commercial or financial relationships that could be construed as a potential conflict of interest.

## Publisher’s Note

All claims expressed in this article are solely those of the authors and do not necessarily represent those of their affiliated organizations, or those of the publisher, the editors and the reviewers. Any product that may be evaluated in this article, or claim that may be made by its manufacturer, is not guaranteed or endorsed by the publisher.
