# Supplementary material for: Loss of Multiple ABCB Auxin Transporters Recapitulates the Major twisted dwarf 1 Phenotypes in Arabidopsis thaliana
Source: Front Plant Sci. 2022 Apr 21;13:840260. doi: 10.3389/fpls.2022.840260 (PMC9069160; doi:10.3389/fpls.2022.840260)
Supplement: Supplementary file 1 [file Data_Sheet_1.docx]

Supplementary Material

**Supplementary Figure 1. Effects of growth conditions on root twisting in *abcb1/19*.**

**Supplementary Figure 2. ABCB transporter protein sequence homology and conservation.**

**Supplementary Figure 3. Expression profiles of *ABCB* transporters and *TWD1*.**

**Supplementary Figure 4. Shoot phenotypes of *abcb4* and *abcb21* higher order mutants.**

**Supplementary Figure 5. Rosette leaves of *abcb1, 19, 6, and 20* mutants.**

**Supplementary Figure 6. Cantil defects in *abcb1/19* mutants.**

**Supplementary Figure 7. Phenotypes of *abcb1/19/20* triple mutants.**

**Supplementary Figure 8. Genetic analysis of *abcb11* mutants.**

**Supplementary Figure 9. Root waving phenotypes of *abcb11-1*.**

**Supplementary Figure 10. Competition of auxin transport with malate in *abcb* mutant seedlings.**

**Supplementary Table 1. Primers used in this study.**

**Supplementary Table 2. Mass transitions from LC-MS/MS quantitations.**

**Supplementary Table 3. Mutant alleles used by figure and table.**


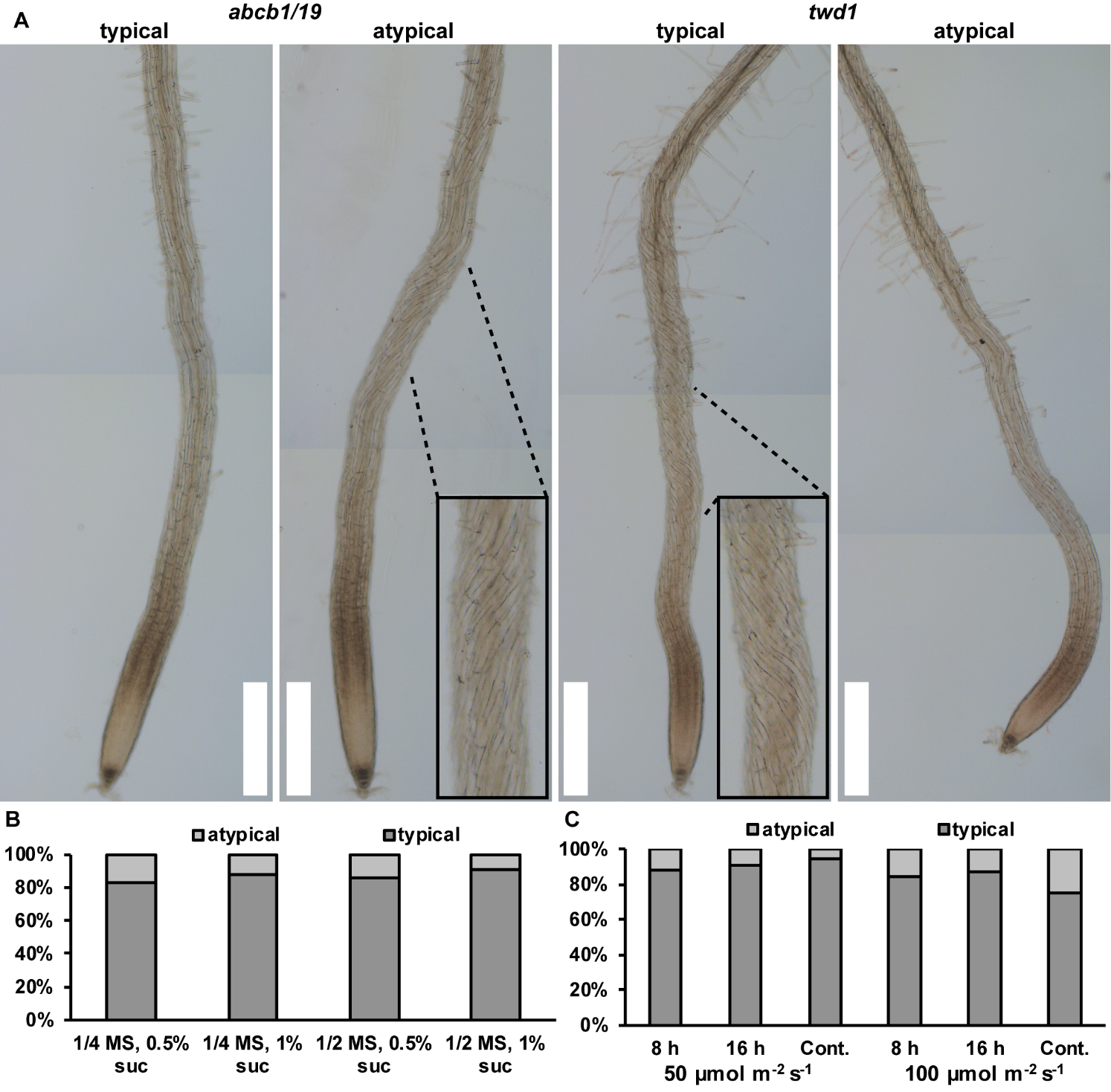


**Supplementary Figure 1. Effects of growth conditions on root twisting in *abcb1/19*.** (**A**) Typical and atypical twisting patterns observed in 7-day *abcb1/19* and *twd1* roots grown on ¼ MS medium containing 1 g L^-1^ MES, 0.5% sucrose, and 0.8% agar (pH 5.6) under continuous 100 µmol m^-2^ s^-1^ light at 22°C. *abcb1/19*. Typical growth patterns in *abcb1/19* consisted of increased root waving and skewing but lacked observable *twd1*-like twisting. Atypical patterns in *abcb1/19* were identified as roots with segments of root twisting that continued for >500 µm (inset). Typical *twd1* roots exhibited continuous cell file twisting while atypical patterns showed segments <500 µm with no twisting present. (**B**) Percentage of *abcb1/19* roots showing typical and atypical growth with altered MS and sucrose concentrations. (**C**) Percentage of *abcb1/19* roots showing typical and atypical growth with altered photoperiod and light fluence. Scale bars: 500 µm.


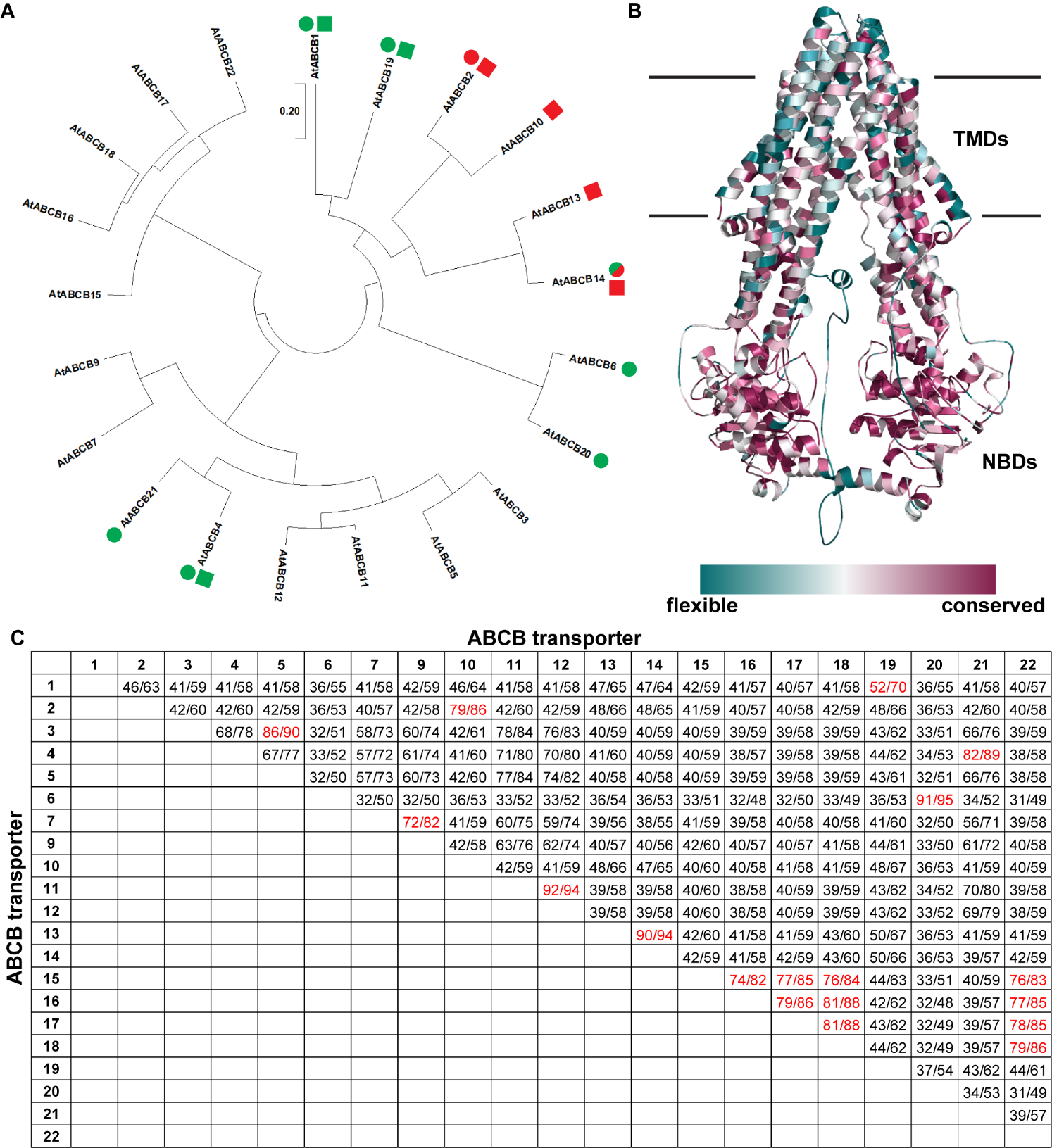


**Supplementary Figure 2. ABCB transporter protein sequence homology and conservation.** (**A**) Relatedness of Arabidopsis ABCB protein sequences by Maximum Likelihood method. The tree is drawn to scale, with branch lengths measured in the number of substitutions per site. Analysis involved 21 amino acid sequences, excluding the pseudogene *ABCB8*. Experimentally verified auxin and non-auxin transporters are indicated by green and red circles, respectively. ABCB14 is a malate transporter but differences in auxin transport have also been reported in *abcb14* mutants. Experimentally determined TWD1 interactors and non-interactors are indicated by green and red squares, respectively. (**B**) AlphaFold2 predicted structure of ABCB19 with colors representing relative amino acid conservation amongst the 21 expressed ABCB proteins. Approximate position of the plasma membrane is indicated by black lines. Sequence variability is primarily associated with the transmembrane domains (TMDs), while the high sequence conservation is associated with the nucleotide binding domains (NBDs). (**C**) Pairwise protein sequence identity and similarity percentages (identity%/similarity%). Red indicates protein sequence conservation within paralogs and paralogous sets. ABCB19 shares the lowest conservation to any other transporter including its paralog ABCB1 (52%/70%). This likely contributes to its high auxin (indole-3-acetic acid) specificity while other ABCBs exhibit broader specificity or transport alternate substrates.


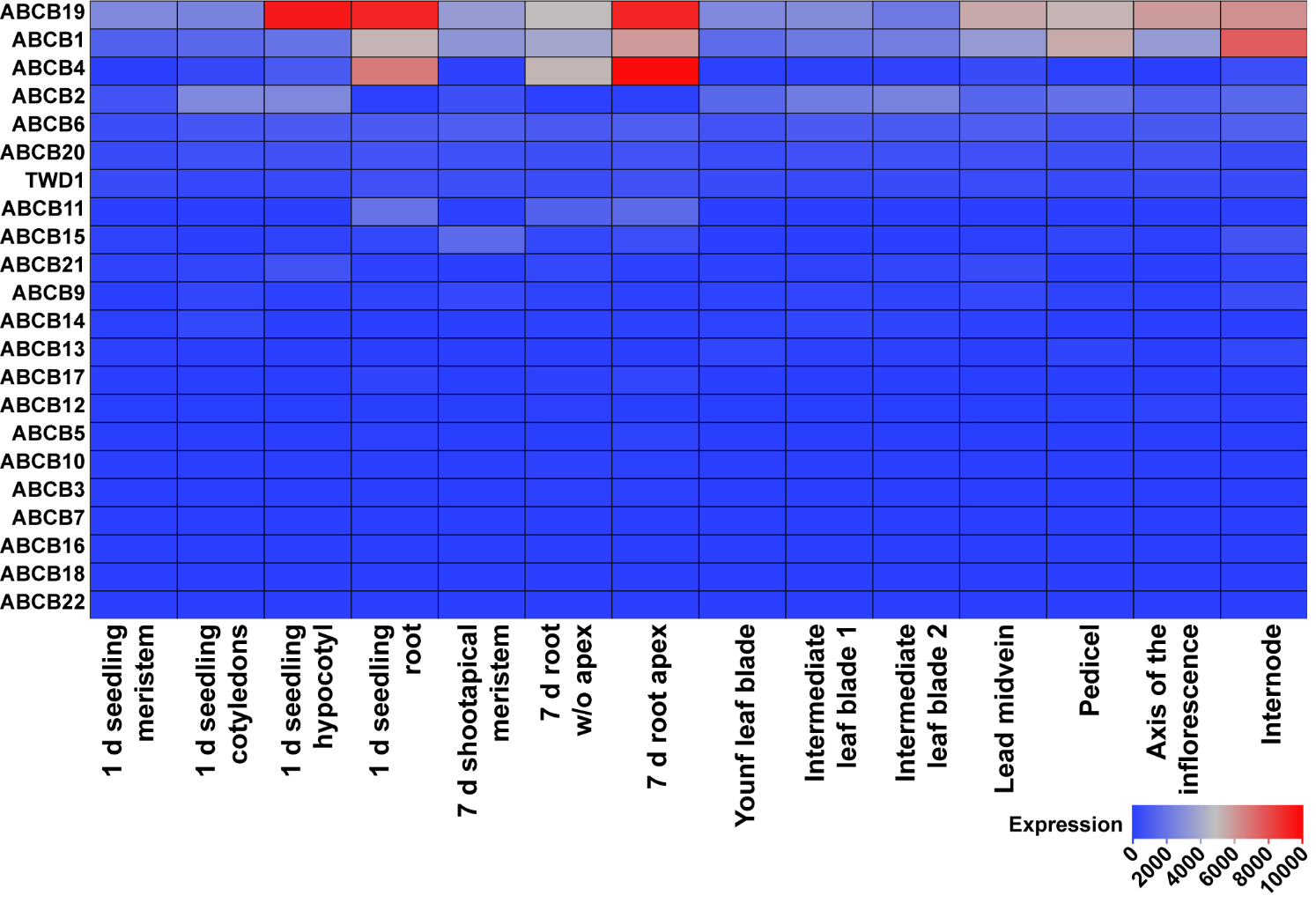


**Supplementary Figure 3. Expression profiles of *ABCB* transporters and *TWD1*.** Heat map showing expression of *ABCB*s and *TWD1* in seedlings, roots, leaves, and inflorescence stems. Genes are presented in descending order based on overall expression (total read number of combined tissues). Data were acquired from the Trava RNAseq database (Klepikova et. al., 2016).


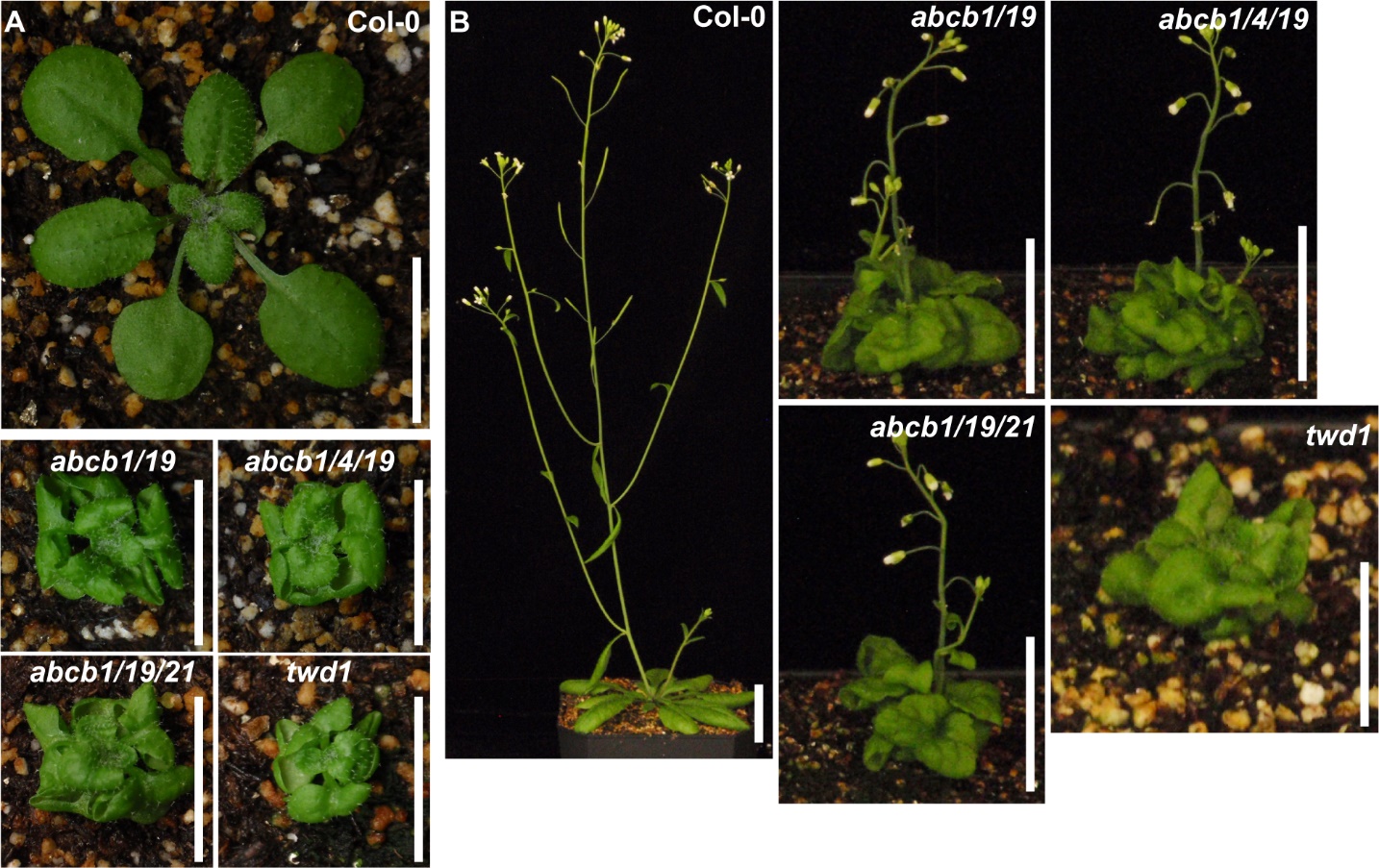


**Supplementary Figure 4. Shoot phenotypes of *abcb4* and *abcb21* higher order mutants.** (**A**) 18-d rosettes of Col-0, *abcb1/19*, *abcb1/4/19*, *abcb1/19/21*, and *twd1*. **(B)** 32-d rosettes and inflorescences of Col-0, *abcb1/19*, *abcb1/4/19*, *abcb1/19/21*, and *twd1*. Scale bars: (A) 1 cm; (B) Col-0: 5 cm, triple mutants: 2 cm, *twd1*: 1 cm.


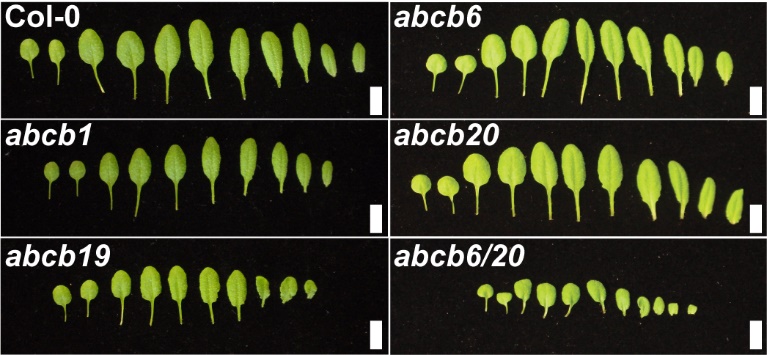


**Supplementary Figure 5. Rosette leaves of *abcb1, 19, 6, and 20* mutants.** Leaves imaged 21 days after germination. Scale bars: 1 cm.


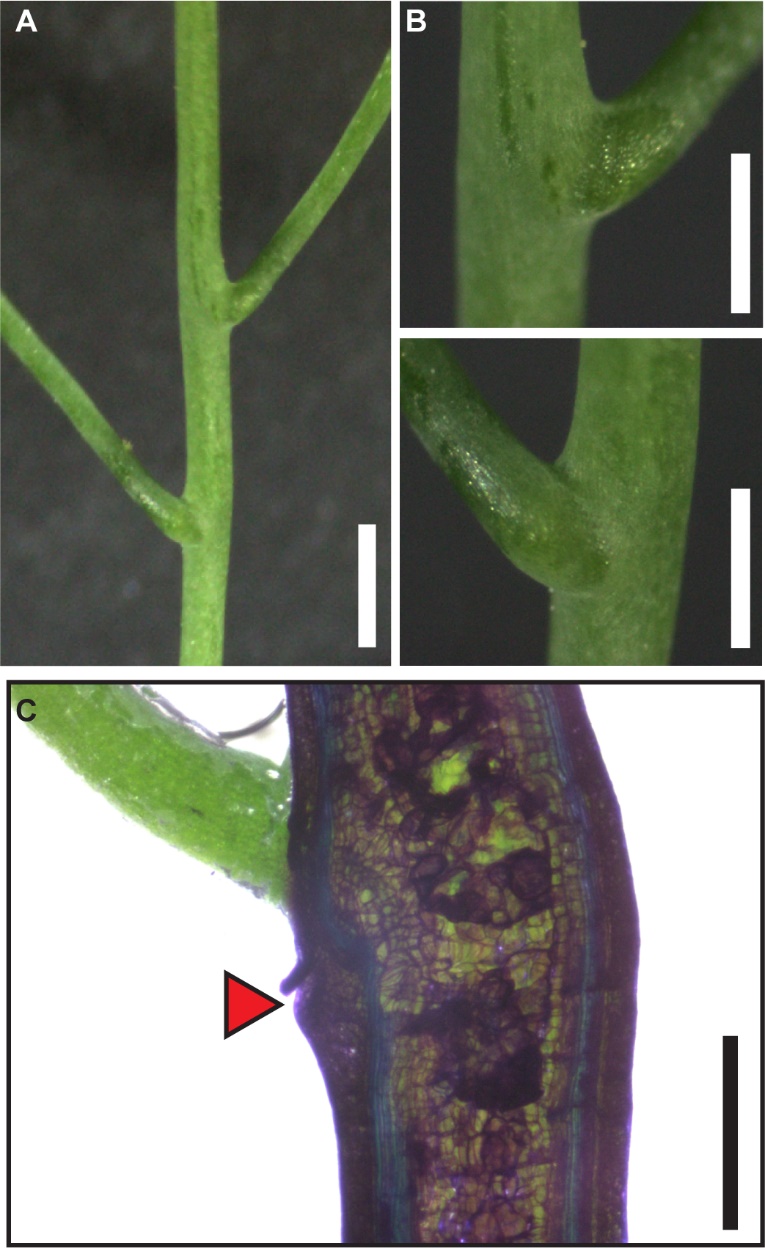


**Supplementary Figure 6. Cantil defects in *abcb1/19* mutants.** (**A**) Cantil cuff formation in *abcb1/19* grown under 100 µmol m^-2^ s^-1^_,_ 16 h photoperiod. (**B**) Close ups of upper and lower cantils in (A). (**C**) Toluidine blue O staining of hand sectioned *abcb1/19*. Cuff outgrowth on the bottom side of the pedicel-stem junction is indicated with the red arrow. Scale bars: (A) 1 mm; (B,C) 0.5 cm.


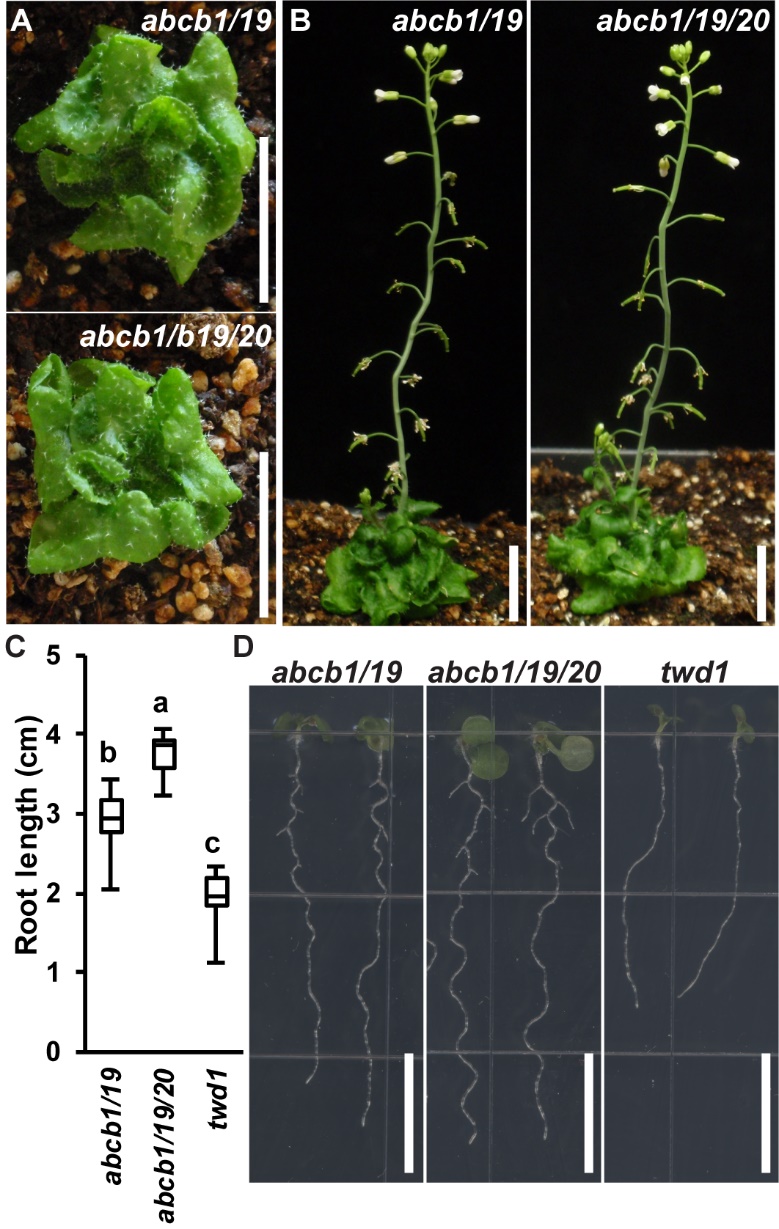


**Supplementary Figure 7. Phenotypes of *abcb1/19/20* triple mutants.** (**A**) Pre-bolting rosettes of *abcb1/19* and *abcb1/19/20*. (**B**) 35-d rosettes and inflorescences of *abcb1/19* and *abcb1/19/20*. (**C**) Quantification of root growth in 7-d seedlings. (n=10-13). Asterisks indicate statistical differences by all pairwise Student’s *t*-test (*, *P* < 0.05). (**D**) Representative images of seedling roots in (C). Scale bars: 1 cm.


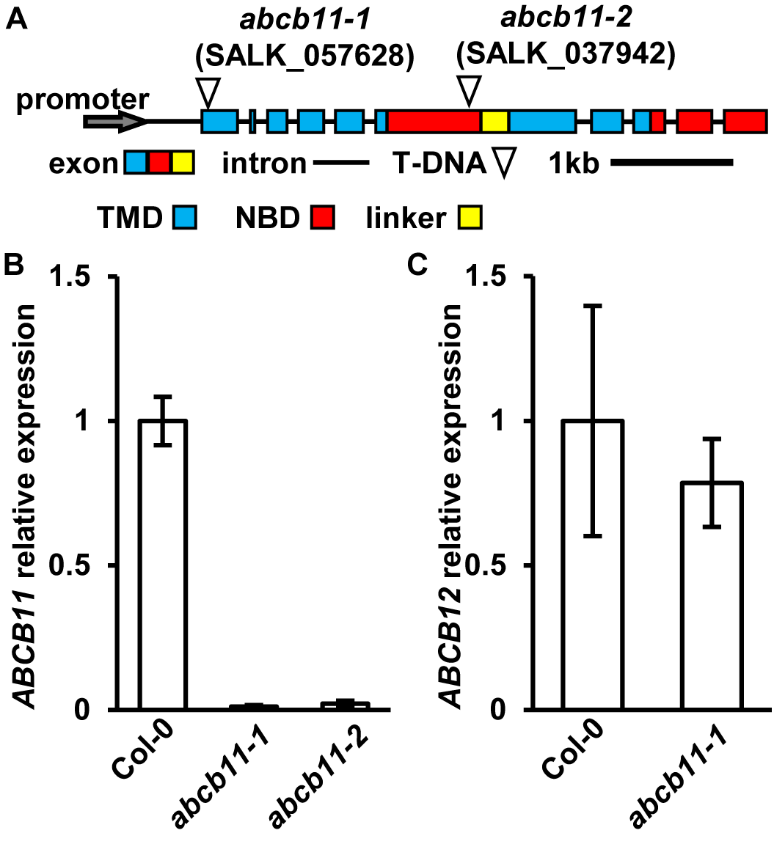


**Supplementary Figure 8. Genetic analysis of *abcb11* mutants.** (**A**) Map of the *ABCB11* locus. Approximate T-DNA insertion positions for *abcb11-1* (SALK_057628) and *abcb11-2* (SALK_037942) within exon 1 and exon 6, respectively, are indicated by white triangles. (**B-C**) Expression of (**B**) *ABCB11* and **(C)** *ABCB12* in *abcb11-1*. RNA was isolated from 5.5-d roots. Data shown are means ± SD (n = 3 biological replicates, 2 technical replicates).


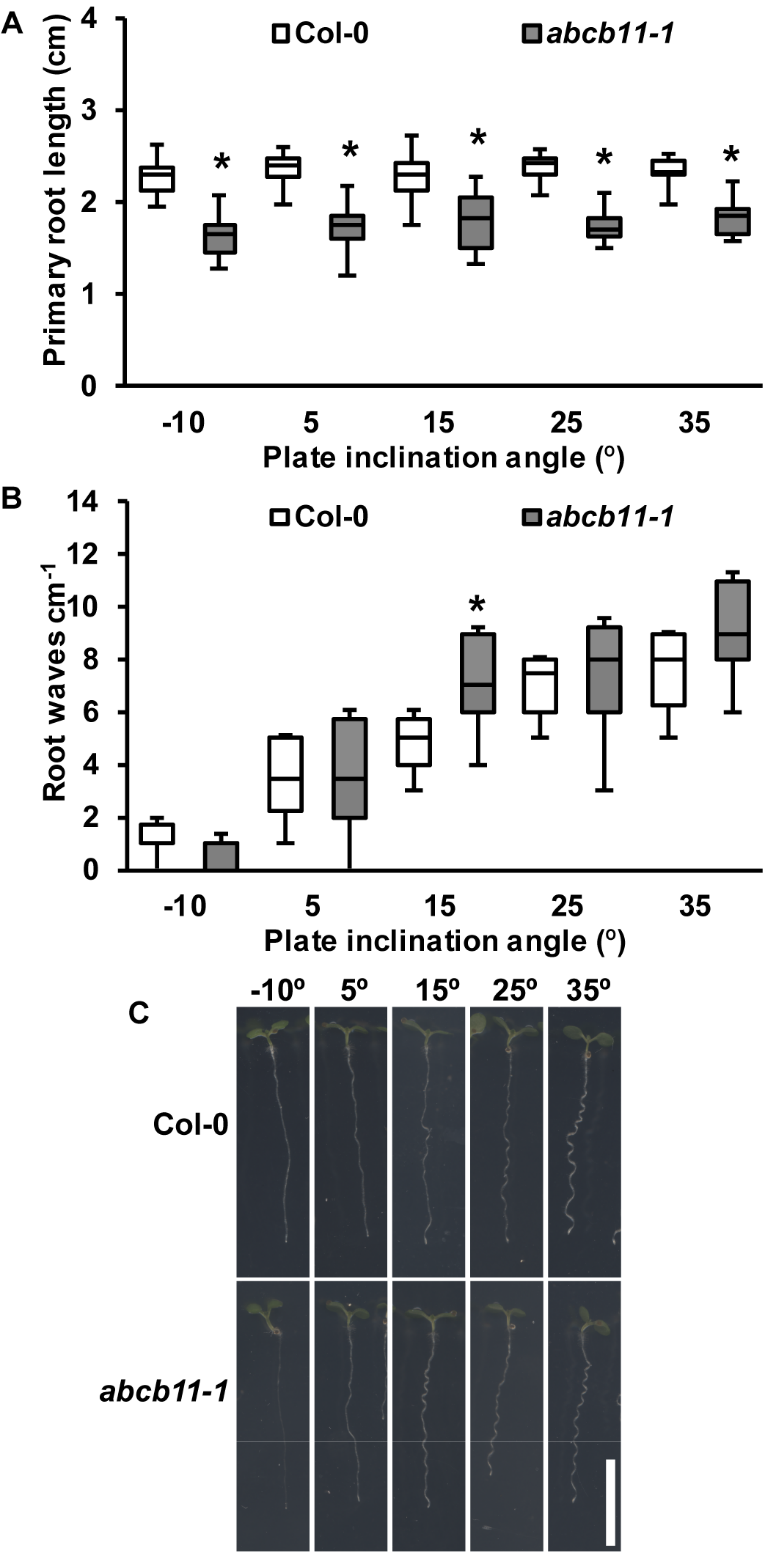


**Supplementary Figure 9. Root waving phenotypes of *abcb11-1*. (A)** Primary root lengths of 5.5-d Col-0 and *abcb11-1* seedlings grown on inclined plates. Angles are degrees from vertical. (n=12-15) **(B)** Quantification of root waving in seedlings from (A). Waves were defined as full oscillations across a vertical line drawn down the root axis. (n=12-15) **(C)** Representative images of Col-0 and *abcb11-1* from (A) and (B). Asterisks indicate statistical difference from Col-0 by Student’s *t*-test (*, *P* < 0.05). Scale bar: 1 cm. All images in (C) are to scale.


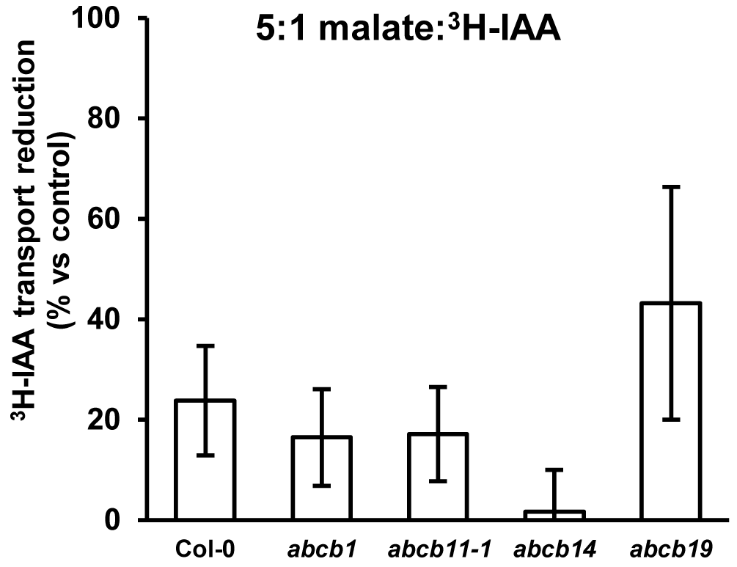


**Supplementary Figure 10. Competition of auxin transport with malate in *abcb* mutant seedlings.** Competition of ^3^H-IAA from the shoot apex to the root-shoot transition zone (RSTZ) in 5.5-d seedlings with a 5:1 molar ratio cold malate. Values represent percent reduction compared to non-competed ^3^H-IAA transport in Figure 7C. Data shown are means ± SD (n = 3 pools of 10). Since ABCB19 is a primary mediator of polar auxin transport, the large error bars in *abcb19* are due to changes in a severely reduced auxin transport background.

**Supplementary Table 1. Primers used in this study.**

| **Quantitative real-time PCR** | | |
| --- | --- | --- |
| **Gene** | **5’-Sequence-3’** | **Sense** |
| *ABCB11* | CGCAGCTCATTCGATTACAAG  ACGAAGTTCCCTCCATTGAC | For.  Rev. |
| *ABCB12* | TGCTTCCCTTGTTCAGCTTCA  GCTACATAAACTTGCATACAACAACATAA | For.  Rev. |
| *PP2A* | TCGTGGTGCAGGCTACACTTTC  TCAGAGAGAGTCCATTGGTGTGG | For.  Rev. |
| *ACT2* | ACACTGTGCCAATCTACGAGGGTT  ACAATTTCCCGCTCTGCTGTTGTG | For.  Rev. |
| **Promoter:GUS Constructs** | | |
| **Construct** | **5’-Sequence-3’** | **Sense** |
| *ABCB11* | CACCTGGACCCTCATGTTTTTCCTT  ATTTCGGCGCTGACAAAAATCAG | For.  Rev. |
| **Expression in *S.pombe*** | | |
| *ABCB11* | ATCATATGATGAACGGTGACGGCGCCAGAGAAG  ATCCCGGGTCAATTAGAAGCAGTCATGTGAAGCTG | For.  Rev. |
| **Genotyping** | | |
| *abcb11-1* | TGGCATCTTGAATAAGAACCG  ATTTTACGGGCAAGCAAAAAG | For.  Rev. |
| *abcb11-2* | AACATCTCCATGTGTAACCGC  TCGGGTGAGTGATACTTTTGG | For.  Rev. |
| LBb1.3 | ATTTTGCCGATTTCGGAAC | T-DNA |

**Supplementary Table 2. Mass transitions from LC-MS/MS quantitations.**

| **Molecule** | **Retention time (min)** | **Precursor ion (m/z)** | **Product ion (m/z)** |
| --- | --- | --- | --- |
| oxIAA | 3.95 | 192 | 146 |
|  |  |  | 128 |
| IAA | 4.61 | 176 | 130 |
|  |  |  | 77 |
| ^13^C-IAA | 4.61 | 182 | 136 |
|  |  |  | 81 |

**Supplementary Table 3. Mutant alleles used by figure and table.**

|  | **Label in figure** | **Alleles** |
| --- | --- | --- |
| **Figure 1** | *abcb1/19*  *twd1* | *abcb1-100 abcb19-101*  *twd1-3* |
| **Figure 2** | *abcb1/19*  *abcb1/4/19*  *abcb1/19/21*  *twd1* | *abcb1-100 abcb19-101*  *abcb1-100 abcb4-1 abcb19-101*  *abcb1-100 abcb19-101 abcb21-2*  *twd1-3* |
| **Figure 3** | *abcb1*  *abcb6*  *abcb19*  *abcb20*  *abcb1/19*  *abcb6/20*  *twd1* | *abcb1-100*  *abcb6-1*  *abcb19-101*  *abcb20-2*  *abcb1-100 abcb19-101*  *abcb6-1 abcb20-2*  *twd1-3* |
| **Figure 4** | *abcb1/19*  *abcb6/20*  *amiRNA1334*  *b6-1 b20-1*  *twd1* | *abcb1-100 abcb19-101*  *abcb6-1 abcb20-2*  *amiRNA1334*  *abcb6-1 abcb20-1*  *twd1-3* |
| **Figure 6** | *abcb11-1*  *abcb11-2*  *abcb1/11-1/19*  *abcb1/19*  *twd1* | *abcb11-1*  *abcb11-2*  *abcb1-100 abcb11-1 abcb19-101*  *abcb1-100 abcb19-101*  *twd1-3* |
| **Figure 7** | *abcb1*  *abcb11-1*  *abcb14*  *abcb19*  *pin1* | *abcb1-100*  *abcb11-1*  *abcb14-1*  *abcb19-101*  *pin1-7* |
| **Supplementary Figure 1** | *abcb1/19*  *twd1* | *abcb1-100 abcb19-101*  *twd1-3* |
| **Supplementary Figure 4** | *abcb1/19*  *abcb1/4/19*  *abcb1/19/21*  *twd1* | *abcb1-100 abcb19-101*  *abcb1-100 abcb4-1 abcb19-101*  *abcb1-100 abcb19-101 abcb21-2*  *twd1-3* |
| **Supplementary Figure 5** | *abcb1*  *abcb6*  *abcb19*  *abcb20*  *abcb6/20* | *abcb1-100*  *abcb6-1*  *abcb19-101*  *abcb20-2*  *abcb6-1 abcb20-2* |
| **Supplementary Figure 6** | *abcb1/19* | *abcb1-100 abcb19-101* |
| **Supplementary Figure 7** | *abcb1/19*  *abcb1/19/20*  *twd1* | *abcb1-100 abcb19-101*  *abcb1-100 abcb19-101 abcb20-2*  *twd1-3* |
| **Supplementary Figure 8** | *abcb11-1*  *abcb11-2* | *abcb11-1*  *abcb11-2* |
| **Supplementary Figure 9** | *abcb11-1* | *abcb11-1* |
| **Supplementary Figure 10** | *abcb1*  *abcb11-1*  *abcb14*  *abcb19* | *abcb1-100*  *abcb11-1*  *abcb14-2*  *abcb19-101* |
| **Table 1** | *abcb1*  *abcb19*  *abcb1/19*  *abcb6*  *abcb20*  *abcb6/20* | *abcb1-100*  *abcb19-101*  *abcb1-100 abcb19-101*  *abcb6-1*  *abcb20-2*  *abcb6-1 abcb20-2* |
| **Table 2** | *abcb11-1*  *abcb11-2* | *abcb11-1*  *abcb11-2* |
